# Correlated Electrostatic Mutations Provide a Reservoir of Stability in HIV Protease

**DOI:** 10.1371/journal.pcbi.1002675

**Published:** 2012-09-06

**Authors:** Omar Haq, Michael Andrec, Alexandre V. Morozov, Ronald M. Levy

**Affiliations:** 1 BioMaPS Institute for Quantitative Biology, Rutgers University, Piscataway, New Jersey, United States America; 2 Department of Chemistry and Chemical Biology, Rutgers University, Piscataway, New Jersey, United States of America; 3 Department of Physics and Astronomy, Rutgers University, Piscataway, New Jersey, United States of America; University of California San Diego, United States of America

## Abstract

HIV protease, an aspartyl protease crucial to the life cycle of HIV, is the target of many drug development programs. Though many protease inhibitors are on the market, protease eventually evades these drugs by mutating at a rapid pace and building drug resistance. The drug resistance mutations, called primary mutations, are often destabilizing to the enzyme and this loss of stability has to be compensated for. Using a coarse-grained biophysical energy model together with statistical inference methods, we observe that accessory mutations of charged residues increase protein stability, playing a key role in compensating for destabilizing primary drug resistance mutations. Increased stability is intimately related to correlations between electrostatic mutations – uncorrelated mutations would strongly destabilize the enzyme. Additionally, statistical modeling indicates that the network of correlated electrostatic mutations has a simple topology and has evolved to minimize frustrated interactions. The model's statistical coupling parameters reflect this lack of frustration and strongly distinguish like-charge electrostatic interactions from unlike-charge interactions for 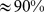 of the most significantly correlated double mutants. Finally, we demonstrate that our model has considerable predictive power and can be used to predict complex mutation patterns, that have not yet been observed due to finite sample size effects, and which are likely to exist within the larger patient population whose virus has not yet been sequenced.

## Author Summary

HIV is incurable because its enzymes evolve rapidly by developing resistance mutations to retroviral inhibitors. Most of these mutations work synergistically, but the biophysical basis behind their cooperation is not well understood. Our work addresses these important issues by bridging the gap between the statistical modeling of HIV protease subtype B sequences with the energetics of mutations involving charged amino acids by showing that electrostatic stability is intimately related to correlations. Moreover, we demonstrate that our statistical model has considerable predictive power and can be used to predict complex mutation patterns that have not yet been observed due to the finite sizes of the current sequence databases. In other words, as the database size increases, our model has the ability to predict the identities of the high probability mutations patterns, which are more likely to be observed. Knowing which currently unobserved mutations are more likely to be observed can be very advantageous in combating the disease.

## Introduction

Proteins evolve through random mutagenesis and their evolutionary selection is constrained by structural, functional and environmental factors [1]. Thermodynamic stability is by far the most important structural factor, as most proteins need to be folded in order to function. The stability range for each protein, however, is narrow and is estimated experimentally to be around 10 kcal/mol, which is of the order of three hydrogen bonds [2]. As a result of this marginal stability, proteins operate “on a knife's edge” [3], whereby a single highly deleterious mutation could potentially lead to decreased stability and loss of activity [4]. By the same token, a single stabilizing mutation could be advantageous from an evolutionary point of view. For example, more stable forms of cytochrome P450 allowed for greater exploration of mutational space in directed evolution experiments than sequences without stabilizing mutations [5]. This increased “evolvability” is not just limited to directed evolution experiments, but may be a general property of proteins evolving under selective pressure [6]. In fact, recent experimental work on HIV protease has shown that accessory mutations compensate for the loss of stability due to destabilizing primary drug resistance mutations, helping the virus evade drugs [7]. This stabilizing effect can have an external source as well: Hsp90, a molecular chaperone, buffers deleterious mutations, allowing for polymorphisms to appear and new traits to evolve [8]. As a result of this work and prior research by other groups, it is now widely recognized that thermodynamic stability is intimately linked with the evolvability of a protein [9]–[11].

Even though the process of mutagenesis is random, the genetic and structural constraints mentioned above, coupled with functional selection, ensure that certain mutations in evolving proteins are associated with each other in a highly non-random fashion [12]. These correlated mutations are an inherent property of evolving amino acid sequences, and an evolutionary signature of viable proteins. A multitude of methods have been developed to identify such pairs and groups of mutations [13], some of which have been applied to HIV protease sequences to locate pairs or groups of coevolving residues [14]–[16]. Our previous work on higher-order correlations showed that for HIV-1 protease, including at least pair correlations is essential for reproducing statistical patterns of primary and accessory mutations observed in protease sequences from patients undergoing anti-retroviral therapy [17].

It is tempting to attribute sequence correlations to effects arising from protein stability constraints [18], and several groups have tried to connect sequence correlations with protein energetics on a detailed atomic level. For example, Zhang et al. applied Bayesian networks to infer therapeutically relevant and conditionally dependent sets of resistance mutations in HIV protease and reverse transcriptase and then used molecular simulations to model the specific interactions that cause resistance [19]. Ranganathan et al. have attempted to explain mutational coevolution by connecting statistical free energies from multiple sequence alignments to differences in experimental folding free energies [20]. Some of these results have been difficult to replicate [21], and are still a topic of active debate in the community [22], [23]. Thus while studies that link mutational correlations to thermodynamic constraints have made great progress [12], [19], [24]–[26], a consensus linking protein energetics and mutational correlation patterns has not yet emerged. These observations have motivated us to explore how correlated mutations in HIV protease are connected to energetics via their impact on protein stability.

Since current methods for predicting stability changes upon mutation based on detailed atomic models are not sufficiently accurate [27], we have chosen to focus instead on the electrostatic part of the total energy for which a coarse-grained model of electrostatics is appropriate. We find that this model captures many important effects of mutations on energetics and stability of HIV protease. We show that the average electrostatic stabilization of HIV protease increases with the number of electrostatic mutations (an electrostatic mutation changes the charge of that mutating residue relative to the wild-type residue), consistent with the hypothesis that accessory electrostatic mutations buffer the destabilizing effects of primary drug-resistance mutations, most of which are non-electrostatic mutations and are therefore not modelled here. We demonstrate that correlations among electrostatic mutations are critical for stabilization; uncorrelated mutations would strongly destabilize the protein. We show that our method, which employs both electrostatic calculations and sequence analysis based on statistical inference techniques, can be used as a predictive tool for novel mutational patterns that have not yet been observed. Finally, we comment on the structure of the electrostatic mutation network of HIV protease. Energy landscape theory, which provided the framework for understanding protein folding through funnels, introduced the concept of a smooth, minimally frustrated landscape for foldable, natural proteins [28], [29]. Our results indicate that the electrostatic interaction network is minimally frustrated as is evident in the derived statistical coupling parameters which strongly predict the underlying charge patterns, providing additional evidence that proteins have evolved to minimize frustrated interactions.

## Results

### Effect of electrostatic mutations on protein stability

**Figure 1:**
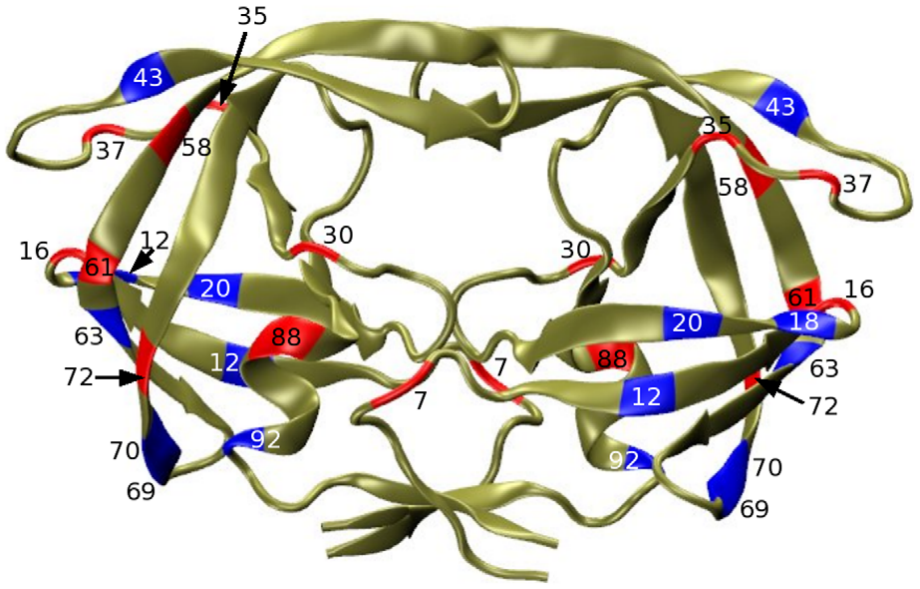
Structure of HIV protease subtype B. The backbone structure of HIV protease subtype B (PDB ID: 1NH0) is depicted in ribbon format. The 18 electrostatically active residues are highlighted. Residue positions which have a predominantly negatively charged non-neutral residue in the sequence database are depicted in red. Residues which have a predominantly positively charged non-neutral residue in the database are depicted in blue.

Our analysis of electrostatic mutation patterns is based on the alignment of 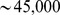 HIV protease sequences from Christopher Lee's HIV Positive Selection Mutation Database (http://bioinfo.mbi.ucla.edu/HIV) [30]. Each amino acid sequence in the Lee database is converted into a charge signature, which is a three letter alphabet representation of that sequence (+, −, n) corresponding to positively-charged, negatively-charged, and neutral residues. These charge signatures are compared to the wild-type charge signature to determine electrostatic mutations. We examined all primary, accessory and polymorphic drug resistance mutation positions (as designated by the Stanford HIV database [31]) and limited our analysis to a subset of 18 positions whose charged state mutates above a threshold frequency of 0.01%. Our model therefore includes more than 380 million states or unique charge signatures involving these 18 positions (Figure 1). Of the 18, 9 are sites which have been characterized as primary or accessory drug resistance mutations while the rest are sites labeled as polymorphic mutations. Mutations are labelled “polymorphic” if they are observed to mutate in the absence of drugs and whose compensatory effect has not yet been experimentally verified, even though drugs may have a significant affect on their correlations with other mutating residues [31].

**Figure 2:**
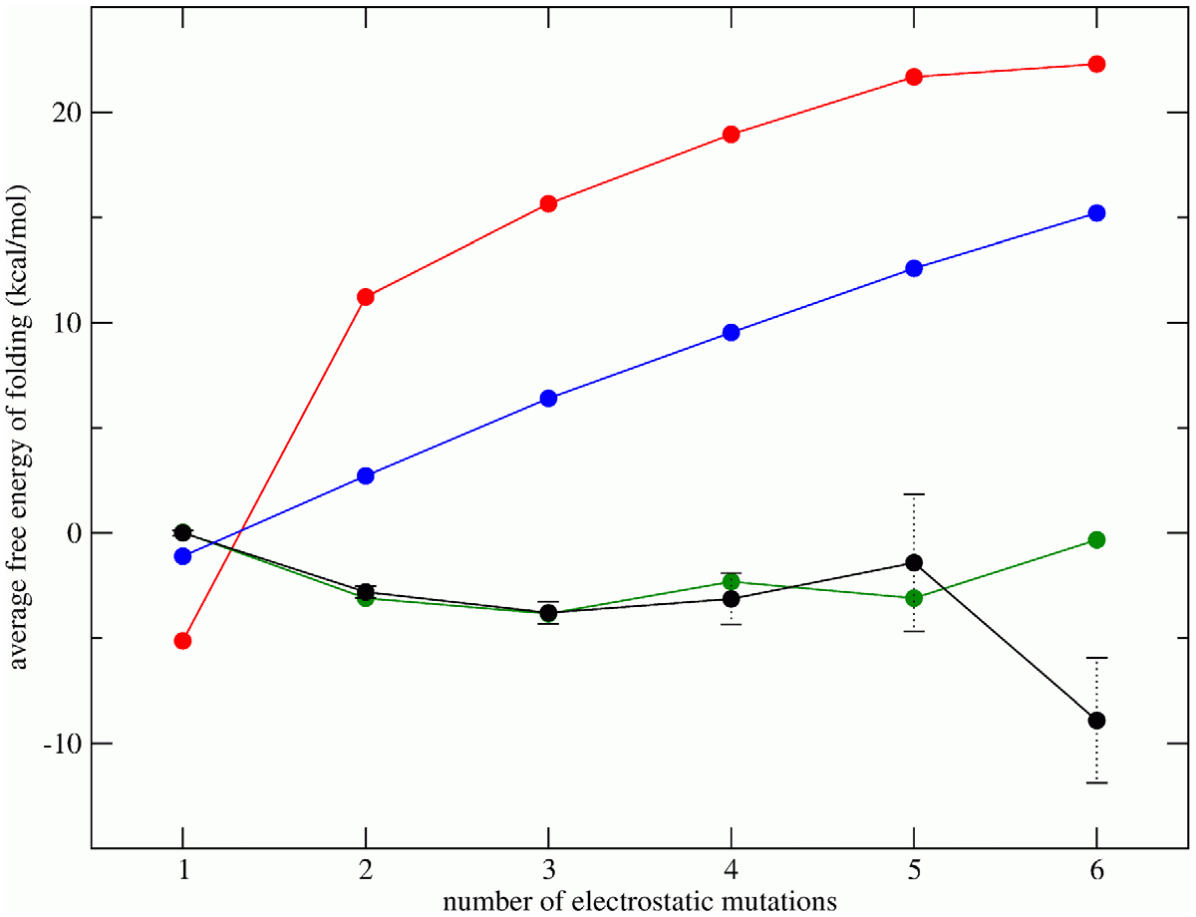
Average electrostatic free energy of folding as a function of the number of electrostatic mutations. Each point on a curve corresponds to 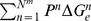, where 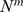 is the number of sequences with 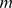 electrostatic mutations, 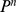 is the probability of the 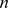
th sequence under a given model conditional upon the number of mutations, and 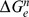 is its electrostatic folding energy (Equation (1); see Methods). All points are plotted relative to 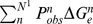, the average 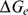 of observed sequences with one electrostatic mutation. The black curve shows the average energies of observed sequences 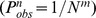, the red curve represents the average energies of sequences under a model in which each charge state occurs with equal frequency, the blue curve shows the average energies of sequences under a model in which each charge state occurs with frequencies observed in the data, and the green curve represents the average energy of sequences under a pair correlation model which preserves observed pair frequencies. The error bars on the black curve are the standard errors of the mean of observed sequences. Note that 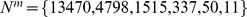 for 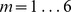.

If we divide this database of charge signatures into subsets with 1, 2, 3… electrostatic mutations and calculate the electrostatic contribution to the average folding free energy 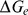, for each subset, we find that on average the stability of the folded state increases by 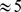 kcal/mol from 1 to 3 mutations and maintains this level of stabilization beyond 3 mutations (Figure 2, black curve). Since selective pressure in the presence of inhibitors often leads to destabilizing primary drug resistance mutations [32], [33], the observed increase in electrostatic stability is due to energetic compensation: destabilizing mutations occur due to selective pressure and electrostatically active residues provide a “reservoir of stability”.

The observed stabilization requires not only the correct frequencies of occurrence of each of the three possible charge states at each position, but also the presence of correlations. Generating random sequences with equal mutation frequencies for the three charge states results in a substantial destabilization of the protein (Figure 2, red curve). Introducing observed frequencies of occurrence of each charge at every position improves the stabilization relative to the previous model with equal mutation frequencies, but still results in substantial destabilization (Figure 2, blue curve). We refer to this latter model as the independent model as it generates an alignment in which mutations at each position occur independently with correct frequencies.

If pair correlations are introduced by preserving the observed joint mutation frequencies (see Methods), substantial protein stabilization occurs, and the energies predicted by this pair correlation model (Figure 2, green curve) become comparable to the energies of the observed sequences. The magnitude of the difference between the observed and pair correlation model average energies is less than 2 kcal/mol for sequences with 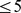 mutations, suggesting that introducing pair correlations is sufficient for explaining the observed energetic stabilization trends. Overall, the difference between the independent model and the pair correlation model is statistically significant given a sample size corresponding to the numbers of sequences observed in the database with those number of mutations (e.g. 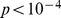 for sequences with 4 or fewer mutations).

### Contribution of specific sequences to the average protein stability and significant drug associations

We find that the observed electrostatic stabilization can be attributed to a relatively small number of low-energy signatures which are highly unlikely under the independent model but become very probable once pair correlations are introduced (Figure S1). For example, the well-studied pair of primary drug resistance mutations, D30N-N88D [15], [32], which occurs 2220 times in the Lee database, contributes 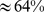 to the 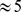 kcal/mol stabilization of the pair correlation model relative to the independent model shown in Figure 2. Together, these top 10 double mutants account for 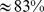 of the stabilization of the pair correlation model relative to the independent model. Table S3 lists the most statistically deviated pairs and Figure S7 depicts the distance between the pairs on the structure of HIV protease. It is interesting to note that 4 out of the top 10 most correlated pairs are greater than 10 Angstroms apart.

With increasing numbers of mutations however, the stabilization spreads among multiple patterns (Figure S1). For 3 electrostatic mutations, the top contributor D30N-N37D-N88D is responsible for 20%, while the top 10 signatures account for 64% of the 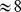 kcal/mol stabilization of the pair correlation model relative to the independent model. For 4 mutations, K20I-D30N-N37D-N88D accounts for 10%, while the top 10 signatures account for 33% of the stabilization. For 5 mutations, K20I-D30N-E35Q-N37D-N88D accounts for 17% and the top 10 signatures account for 36% of the stabilization.

These stabilizing charge patterns are also strongly associated with protease inhibitor therapies, as determined by our drug association analysis (see Text S1). Most protease drug association studies focus on point mutations or pairs of mutations [30], [34]. Our drug association analysis allows us to examine the significance of drug association for patterns of more than two mutations. Table S1 lists the most significant associations between drugs and charge patterns of 2, 3 and 4 electrostatic mutations with the highest probabilities as predicted by the pair correlation model. Most of the patterns listed are strongly associated with at least one drug and several are associated with many drugs. For example the D30N-N88D double mutant and the D30N-N37D-N88D triple mutant are both strongly associated with Nelfinavir monotherapy and Indinavir-Nelfinavir combination therapy with 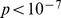. We also find strong association between drugs and patterns predicted by the pair correlation model with more than three mutations. For example K20I-D30N-N37D-N88D and K20I-D30N-E35Q-N88D are both associated with Indinavir-Nelfinavir combination therapy while K20I-D30N-H69Q-N88D is associated with Ritonavir-Nelfinavir therapy with 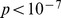.

### Predicting novel mutational patterns

The ability to predict drug resistant mutation patterns is of great therapeutic relevance. Approaches to predicting drug resistance mutations based on biophysical modeling have recently been proposed [35], [36]. In contrast, our statistical-inference based approach which includes pair correlations among mutations, allows us to predict the probabilities of arbitrary charge signatures, many of which have not yet been experimentally observed. Figure S2 shows that most of the sequences with less than 5 mutations, whose probabilities are significantly enhanced by pair correlations, are observed in the Lee database, indicating that these mutational patterns are routinely utilized by the virus. However, for 6 mutations the most stabilizing pattern, K20I-D30N-E35Q-N37D-Q58E-N88D, was not observed (Figure S1). The probability of this pattern under the pair correlation model is 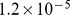, too small to appear frequently in a database of 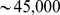 sequences due to finite sample size effects. However, if the size of the database were to increase five-fold, the probability of observing at least one copy of this pattern would be 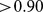.

**Figure 3:**
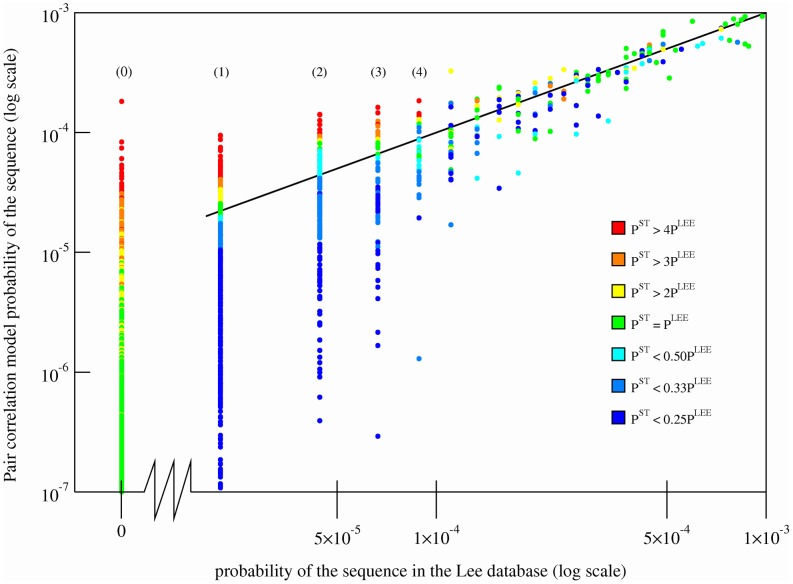
Comparison of the sequence probabilities in the tail of the Lee database and the pair correlation model with the sequence probabilities in the Stanford database. The probabilities of sequences under the pair correlation model, 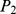, predicted using the Bethe approximation, are plotted as a function of the sequence probabilities from the Lee database, 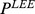. Sequences with a probability of 0 in the Lee database, i.e. unobserved sequences, are plotted to the left of the abscissa break. Every sequence is shaded using a color gradient corresponding to 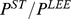, which represents the number of times the sequence occurs in the Stanford database, relative to its probability in the Lee database. Sequences that occur frequently in the Stanford database as compared to the Lee database have a higher ratio and are shaded red, while the sequences that do not occur as frequently in the Stanford database as compared to the Lee database have a lower ratio and are shaded blue. Sequences that are shaded green have equal probabilities in both databases. Sequences unobserved in the Lee database (leftmost row in the graph), but observed in the Stanford database have a ratio that is artificially set to equal 4, which corresponds to the color red. Unobserved Lee sequences that are also unobserved in the Stanford database are shaded green because 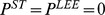. Sequences with probabilities 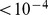 are shaded according to the average value of 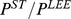 for a window of 10 sequences around the sequence of interest. Sequences with probabilities 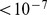 or 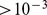 are not shown. The indices (0), (1), (2), etc mark the locations of sequences observed zero, once, twice (etc) in the Lee database. Each dot corresponds to a unique sequence.

The proportion of sequences not observed in the Lee database with significantly enhanced pair correlation model probabilities increases greatly with the number of mutations, of which it is likely that many are not observed because of finite sample size effects (Figure S2). In order to test our ability to predict novel patterns of favorable electrostatic mutations unobserved in the Lee database due to finite sample size effects, we examined the contents of a separate database, the drug-annotated Stanford database which contains HIV protease subtype B sequences from various sources [31]. Figure 3 plots the probabilities of sequences using the pair correlation model, 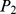, as a function of the observed probabilities in the Lee database. Sequences are also shaded according to a gradient that represents how often the sequence occurs in the Stanford database, relative to the Lee database. The plot shows that sequences with the highest predicted 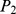 probabilities that are unobserved in the Lee database are largely shaded red, indicating that most are observed in the Stanford database. In fact, of the top 25 most probable sequences predicted by the pair correlation model that are not found in the Lee database, 19 are present in the Stanford database (Table S2). Most of these sequences are also significantly associated with drug therapies (e.g. 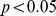). As the predicted 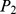 probability decreases, the shading of the dots gradually changes to green, indicating that sequences with the lowest predicted 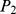 probabilities are unobserved in both the Lee and the Stanford database. Thus our approach exhibits considerable predictive power. If we examine other sequences in the tail of the Lee probability distribution that are observed once, twice, three times etc in the Lee database, we notice a similar trend; sequences with higher predicted 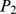 probabilities are present in the Stanford database at much higher frequencies than sequences with lower predicted 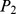 probabilities, even though the pair correlation model was parameterized on the Lee database. This result strongly suggests that the pair correlation model is a much better predictor of actual sequence probabilities than using the sequence counts from the databases themselves, because of finite sample size effects. In other words, the tail of the distribution is very well represented by the pair correlation model.

### Structure of the electrostatic mutation network

Determining the statistical field and coupling parameters (written as 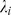 and 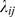 for simplicity) that best fit the pair correlation model given a set of observed univariate and bivariate marginals (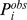 and 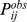), is known in the literature as the inverse Ising problem. As described in the Methods, we iteratively determine these parameters using a graph-theoretic inference algorithm, called belief propagation (BP) [37]–[39], a method which has recently been applied by other research groups to study protein conformational entropy and protein-protein interactions [40], [41]. Within the BP framework, we apply a mean field model which includes pair correlations in the Bethe approximation to estimate the bivariate marginals, 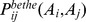 [38], [42], [43]. In the statistical physics community “mean field” in often used to refer to a class of approximations whereby the free energy of the system is written in terms of the marginals up to a given order, the corresponding mean field model at the pair correlation level is the Bethe approximation. However, it is well known that while 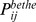 is exact and converges to 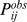 on acyclic networks, 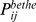 only approximates 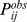 and can become unstable on cyclic networks [44]. For the electrostatic correlation network of HIV protease, we observe that the belief propagation algorithm converges quickly to the observed bivariate marginals (Figure S3). The convergence towards the observed probabilities for triplets and larger multiplets is also well approximated, a result that is non-trivial since the Bethe approximation is a pair-level approximation and does not guarantee the convergence for marginals beyond pairs [45]. For trivariate marginals, the correlation coefficient between 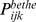 and 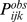 is 0.98 while for four mutations, the correlation coefficient between 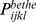 and 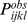 is 0.90 (Figure S3). This close correlation between observed and predicted marginals argues for a simple network structure and suggests that for this system, the Bethe approximation is a good approximation and by implication the electrostatic mutation network is minimally frustrated.

**Figure 4:**
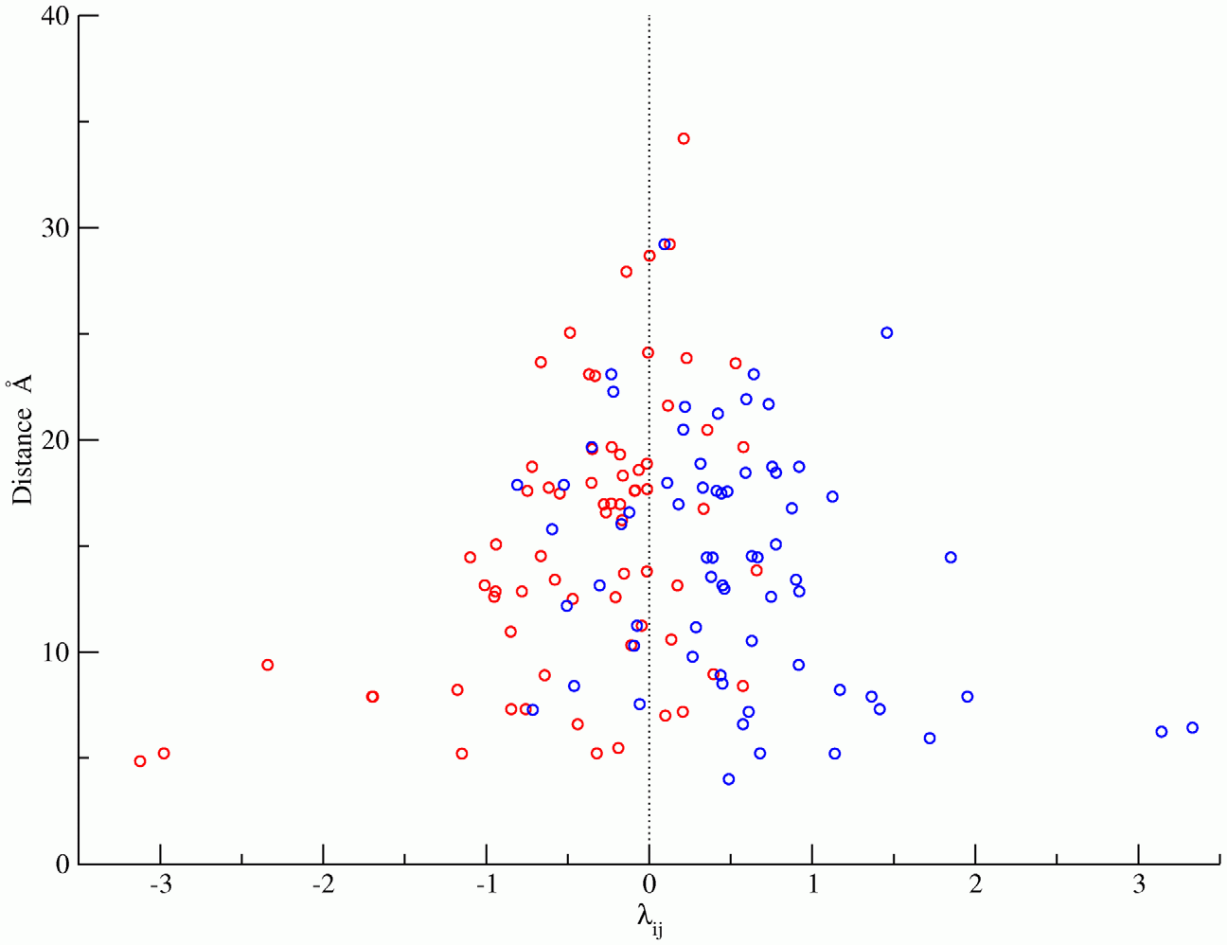
Distance between like and unlike-charge pairs as a function of the statistical coupling parameter, 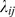. The statistical coupling parameter 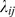 is a fitting parameter that describes the statistical interaction energy between pairs of states. Since the Bethe mean field pair correlation model is a good approximation for this data, a negative 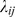 indicates that a pair of states is enhanced (positively correlated), while a positive 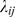 indicates that a pair of states is suppressed (negatively correlated). Using simple electrostatics, we observe that like-charge patterns (blue) are mostly suppressed while unlike-charge patterns (red) are enhanced. The sign of 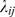 is able to correctly predict the charge patterns for 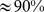 of the top 35 most significantly correlated charge pairs out of a total of 135 pairs. The p-value for the statistical significance of this result is 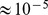. The reason there are 135 pairs is as follows: For each pair of residues, there are 4 possible sets of like and unlike charge combinations, resulting in a total of 612 like/unlike charge pair combinations. However, not all pairs exist in the database or are significantly correlated. Filtering results in 135 pairs with probability greater than 0.001%.

Another strong indicator of the lack of frustration in the electrostatic mutation network of HIV protease is that the statistical coupling parameters of the (Bethe) mean field model are able to distinguish like-charge patterns from unlike-charge patterns with high accuracy (Figure 4). We find that the sign of 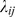, a quantity derived from the sequence analysis alone, is able to correctly predict the charge patterns for 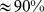 of the top 35 most significantly correlated charge pairs (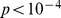.), reflecting the evolutionary optimization of the protein electrostatic interaction network (Figure 4). Moreover, Figure 4 also indicates that the magnitude of 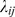 correlates with the spatial distance between residues. Of the top ten pairs of residues with the largest statistical coupling parameters, nine are situated close to one another (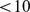 Å) in the folded structure of the HIV protease homodimer. In this context, we note that Morcos et al. [46] used a similar approach to infer spatial contacts between residues of many proteins, through an analysis of the coupling parameters of a corresponding mean field model.

## Discussion

Our results suggest that electrostatic interactions play an important role in the coevolution of mutations in HIV protease. The extent to which electrostatics influences protein stability has been the subject of debate in the literature [47]–[53]. One experimental study of protease has minimized the role of electrostatics in favor of the impact of compensatory mutations on protein flexibility [54]. Others have suggested that buried charges play a more important role in protein function than stability [55], [56]. We recognize that electrostatics is only part of the total energy, and that contributions to stability from van der Waals interactions, hydrogen bonding and hydrophobic effects are significant. Nonetheless, long-range electrostatics is likely to have a substantial effect on protein stability [50], [52], [57]–[59].

Our results support the proposal that the presence of correlations among electrostatic mutations arises from the contraints imposed by the need to maintain the stability of the folded protein. HIV protease is under strong selection pressure from drugs. As a result of the initial build up of drug resistance, protease becomes less stable [32]. We hypothesize that electrostatic mutations not only bring the protein back to a more viable state, but may give the protein more “breathing room” on the evolutionary fitness landscape. Manipulating the charge distribution of HIV protease is complex and we find that uncorrelated mutations would tend to strongly destabilize the enzyme, contrary to the stability gain observed in the database. Therefore, we propose that sets of electrostatic mutations occur together, increasing the “evolvability” of a protein by providing a “reservoir of stability” which allows it to escape epistatic traps along evolutionary pathways towards higher fitness [18].

The absence of frustration could reflect evolutionary optimization of the electrostatic interaction network in HIV protease under selection pressure from drugs, or it could be a general property of protein electrostatic interaction networks. Indeed, natural proteins tend not to be frustrated systems [29], [60] – they are fine-tuned biological machines with restricted evolutionary pathways [18]. Within these pathways, proteins are highly robust and the physics underlying their folding display a kind of simplicity [29], [61]–[63]. Our conclusions based on a coarse-grained electrostatics model combined with statistical inference techniques reflect this lack of frustration. In future work we will study our algorithm on interaction networks with a larger alphabet size and different network topologies. In this context, we note that Balakrishnan et al. used an alternative learning algorithm that a solved similar problem but determined the optimal graph topology for the network [64].

Our statistical analysis of HIV sequences captures biophysical constraints in the form of a statistical network of correlated mutations. Even though the model is based on pairwise correlations, it captures the higher-order effects and correctly predicts the probabilities of sequences found in the tail of the distribution. The fact that many of these patterns are also strongly associated with protease inhibitors from patients undergoing antiretroviral therapy, highlights the clinical relevance of our method. Other mutation patterns that we predict are likely to exist within patients whose virus has not yet been sequenced. Having knowledge of these unique, but as yet unobserved patterns, can be important for the design of future inhibitors to combat drug resistance.

In this work, we go beyond a purely statistical approach to modeling patterns of electrostatic mutations, and show that the statistical results are entirely consistent when viewed in the context of a structure based energy model. Though electrostatics is only part of the total energy, our work has highlighted its importance and provided support for the proposal that correlated electrostatic mutations provide a reservoir of stability for HIV protease as it builds resistance to drugs.

## Methods

### HIV sequence databases

45,161 aligned HIV-1 DNA nucleotide sequences were downloaded from Christopher Lee's HIV Positive Selection Mutation Database (http://bioinfo.mbi.ucla.edu/HIV) on March 4th, 2008 [30]. This database of sequences, which we call the Lee database, consists primarily of HIV-1 subtype B samples sequenced by Specialty Laboratories Inc from 1999 to mid-2002. These sequences are not annotated. [30]. The amino acid sequences were converted into strings of characters “n”, “−” and “+”, indicating whether a given residue is neutral, negatively or positively charged at pH = 6 (i.e. His, Arg, and Lys are positively charged, while Asp and Glu are negatively charged). A second database of subtype B sequences, which we call the Stanford database, was downloaded from the Stanford HIV database on April 7th, 2010 [31]. This database consists of drug annotated sequences collected primarily from more than 900 literature and GenBank references. This drug-annotated dataset was used to associate correlated mutation patterns with specific anti-retroviral therapies (see SI). The univariate and bivariate marginals extracted from the Lee database and from the Stanford database are effectively the same (correlation coefficient of 0.999), indicating that our results would be unchanged if we used the Stanford database to parameterize the model. Moreover, at a 20 letter amino acid alphabet level, there is little redundancy between the databases as only 7.63% of the sequences are present in both databases.

To locate electrostatic mutations, the resulting charge signatures were compared to the HIV-1 subtype B consensus sequence from the Los Alamos National Laboratory HIV sequence database. This consensus sequence was used to define the wild-type charge signature. We define an electrostatic mutation as an amino acid mutation which changes the charge at a certain position along the protein sequence, relative to the wild-type amino acid at that position (e.g. D30N and N88D). In contrast, the L90M and R8K are not considered to be electrostatic mutations.

We examined all the primary, accessory and polymorphic drug resistance mutations positions (as designated by the Stanford database [31]) and included all electrostatic mutations above a threshold frequency of 0.01%. The 18 positions included are the primary drug resistance mutatation sites D30 and N88, accessory mutation sites K20, E34, E35, K43, Q58, L63, and Q92, and polymorphic mutation sites Q7, T12, G16, Q18, N37, Q61, H69, K70, and I72.

### Calculation of electrostatic folding free energies

The electrostatic energy of protein folding 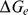 was estimated as
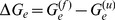
(1)where 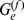 and 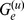 are electrostatic free energies of the folded and unfolded states, computed using an Analytical Generalized Born (AGB) model [65]. The folded state electrostatic free energy 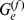 was calculated by placing unit charges corresponding to a particular charge signature onto the most-distal sidechain carbon atom of the corresponding wild-type amino acid within a dimer crystal structure (PDB ID 1NH0). All other sidechain atoms remain neutral, although a partial charge dipole of 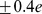 is placed on every backbone amide and carbonyl group to retain the helix dipole effects [66].

Our approximation of the denatured state is a maximally extended structural representation of chain A from 1NH0, with backbone dihedral angles set to 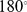 (except for prolines) and sidechain rotamer states set to all-*trans*. Similarly to the folded state, charges on the unfolded state are placed on the most-distal sidechain carbon atom and backbone dipoles are switched on. See SI Methods for further implementation details.

### Statistical modeling of sequence probabilities

As in our previous work [17], we make use of a Potts model to capture the effects of pair interactions between residues. Since our electrostatic data consists of sequences with three possible charge states at each site, we use a 3-letter alphabet (+, −, n), for positively-charged, negatively charged, and neutral residues. Including all three charge states in our study leads to 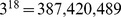 possible charge signatures for 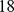 positions.

For each signature we calculate 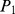, the independent model probability, and 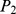, the pair correlation model probability. Specifically, we fit the frequencies of charge states at each position and the joint frequencies of charge states at pairs of positions to the 3-state Potts model:
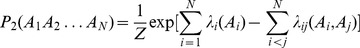
(2)where 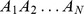 is a sequence of 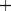
's, 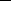
's or 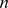
's of length 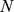, 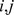 are position indices, 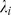 and 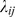 are the fitting parameters for the fields and couplings, and 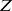 is the partition function. The independent model, obtained by setting all 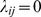, corresponds to 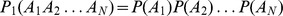. For the equal frequency model in Figure 2, we set 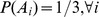.

The joint probability distribution given by the Potts model has the largest entropy constrained by the univariate (independent model) or both univariate (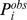) and bivariate (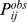) marginals from the data [67]. To solve the inverse Ising problem, we implemented an efficient graph-theoretic inference algorithm called belief propagation (BP) [37]–[39]. Our algorithm employs a two-step procedure: first, all the univariate and bivariate marginals are determined for a given set of 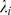 and 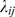 in the Bethe approximation within BP [38], [42], [43]. Second, the predicted marginals are compared to the observed marginals to determine updated 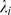 and 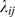 via gradient descent [39]. See the supporting information for further information about the algorithm.

## Supporting Information

Figure S1
**Contribution of individual sequences to the average electrostatic folding energy.** Each contribution is given by 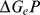, where 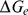 is the electrostatic folding energy of a given sequence (see Methods) and 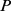 is its probability under the independent or pair correlation model conditional upon the number of mutations. Red: mutation patterns observed in the Lee database [30], black: mutation patterns not observed in the Lee database. Several outliers are labeled explicitly by their mutation pattern. Mutations are represented as 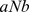, where 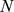 is the residue number and 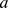 and 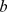 are one of the 3 charged states (+, −, n). The straight line on each diagram is a plot of 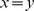. Sequences below this line have 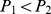, resulting in 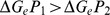 (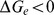). For these sequences, the electrostatic stabilization is greater under the pair correlation model than under the independent model.(TIF)

Figure S2
**Comparison of sequence probabilities under the independent and pair correlation model.** The probability of a given sequence under the pair correlation model, 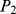, is plotted against the probability of the same sequence under the independent model, 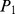, for all sequences with 1 through 6 electrostatic mutations. Both independent and pair correlation model probabilities are renormalized and are conditional upon the number of mutations. Red: mutation patterns observed in the Lee database [30], black: mutation patterns not observed in the Lee database. Several outliers are labeled explicitly by their mutation pattern. Mutations are represented as 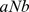, where 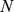 is the residue number and 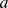 and 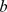 are one of the 3 charged states (+,−, n). The straight line on each diagram is a plot of 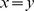. Sequences below this line have 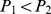.(TIF)

Figure S3
**Comparison between the observed and predicted mutivariate marginals for 2, 3 and 4 mutations.** Predicted marginals determined using belief propagation in the Bethe approximation are plotted against the observed marginals for sets of 2, 3, and 4 mutations. The correlation between 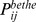 and 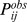 is 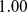. The correlation between 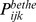 and 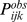 is 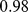. The correlation between 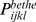 and 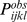 is 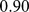.(TIF)

Figure S4
**Distribution of pair correlation model probabilities for sequences in the tail of the Lee distribution that are observed (red) or unobserved (blue) in the Stanford database.** The histogram in red is the distribution of pair correlation model probabilities for sequences found in the tail of the Lee database that also exist in the Stanford database. The histogram in blue is the distribution of pair correlation model probabilities for sequences that are not observed in the Stanford database. The null hypothesis which states that the means of these two distributions are equal, has a low p-value of 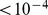, indicating that the null hypothesis must be rejected. Therefore, the difference between the means of these two distributions is statistically significant.(TIF)

Figure S5 **Distribution of the number of sampled sequences not observed in the Lee database.** 13,286 sequences, corresponding to the size of the Stanford database, were randomly sampled from the probability distribution described by the pair correlation model. The distribution of the number of sequences not observed in the Lee database for each of the 1,000 simulations, is plotted as a frequency distribution. The sample average for this distribution is 124.2 and the standard deviation is 10.6. The actual number of sequences in the Stanford database that are not observed in the Lee database is 128 (plotted as a straight red line), a number which lies well within 1 standard deviation of the sample mean.
(TIF)

Figure S6 **Distribution of the number of unique sequences for sample sizes equal to the size of Stanford and Lee databases.** 13,286 and 45,161 sequences, corresponding to the sizes of the Stanford and Lee databases, were each randomly sampled from the probability distribution described by the pair correlation model. The distribution of the number of unique sequences for 1,000 simulations for both sampling distributions is plotted as a histogram. The sample average for the Stanford-sized sample distribution is 452.9 and the standard deviation is 14.3. The sample average for the Lee-sized sample distribution is 862.1 and the standard deviation is 18.7. The number of unique sequences in the Stanford database is 431 while the number of unique sequences in the Lee database is 828, both of which lie within 1.4 standard deviations of their respective sample means.
(TIF)

Figure S7 **Structure of HIV protease subtype B and the spatial distances between highly correlated pairs.** The backbone structure of HIV protease subtype B (PDB ID: 1NH0) is depicted in ribbon format. Similar to Figure 1, the 18 electrostatically active residues are highlighted. Residue positions which have a predominantly negatively charged non-neutral residue in the sequence database are depicted in red. Residues which have a predominantly positively charged non-neutral residue in the database are depicted in blue. Addititionally, the distances between the top 5 most correlated pairs of residues are depicted as dashed lines. The pairs are 30–88, 20–35, 16–63, 18–20 and 20–92.
(TIF)

Table S1
**Electrostatic mutation patterns with the highest probabilities under the pair correlation model and the drug combinations they are most strongly associated with.** Shown are the top 5 patterns with 2, 3 and 4 electrostatic mutations for which the pair correlation model predicted probability, 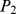, is the highest, together with the drug combination they are most significantly associated with. Drug combinations are listed in order of treatment. The test of statistical association between drugs and electrostatic mutation patterns is based on the the Stanford database [31] (SI Methods). The proportion of sequences with the mutation pattern and exposed to a specific drug was compared to the proportion of sequences with the same mutation pattern but exposed to no drugs. The null hypothesis is that that the two proportions are equal, and the p-value to test the significance of this hypothesis is listed alongside the drug combination. NFV: Nelfinavir, IDV: Indinavir, SQV: Saquinavir, RTV: Ritonavir, APV: Amprenavir. The acronym PI, protease inhibitor, is used in the Stanford database when the drug was unknown. The 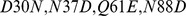 pattern is not significantly associated with any drug combination.(PDF)

Table S2
**Prediction of novel electrostatic mutation patterns.** Shown are 25 electrostatic mutation patterns with the highest probabilities under the pair correlation model that are not observed in the Lee database [30]. 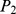 is the probability of the sequence under the pair correlation model, 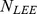 is the number of times the mutation pattern was found in the Lee database [30], 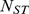 is the number of times the mutation pattern was found in the Stanford database [31]. If the sequence is found in the Stanford database, it may be significantly associated with specific drugs combinations. The drug combinations listed are in order of treatment and have strong p-values of association with the mutation pattern. The test of statistical association between drugs and electrostatic mutation patterns is described in SI Methods. NFV: Nelfinavir, IDV: Indinavir, SQV: Saquinavir, RTV: Ritonavir, APV: Amprenavir. The acronym PI, protease inhibitor, is used in the Stanford database when the drug was unknown.(PDF)

Table S3
**The most statistically deviated pairs of mutations.** The 10 most statistically deviated double mutations in the Lee database relative to the independent model. The measure used to test for deviation is 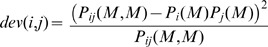 where 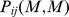 is the joint probability of a double mutation at positions 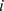 and 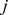 while 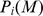 is the univariate marginal of a mutation at position 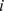. The double mutant charge states and the distance between charges is also listed.(PDF)

Text S1 **Text S1 consists of the supplementary information to the main text.** In this supplementary information, we describe our algorithm in greater detail.
(PDF)
