# Supplementary figures and images for: Correlated Electrostatic Mutations Provide a Reservoir of Stability in HIV Protease
Source: PLoS Comput Biol. 2012 Sep 6;8(9):e1002675. doi: 10.1371/journal.pcbi.1002675 (PMC3435258; doi:10.1371/journal.pcbi.1002675)

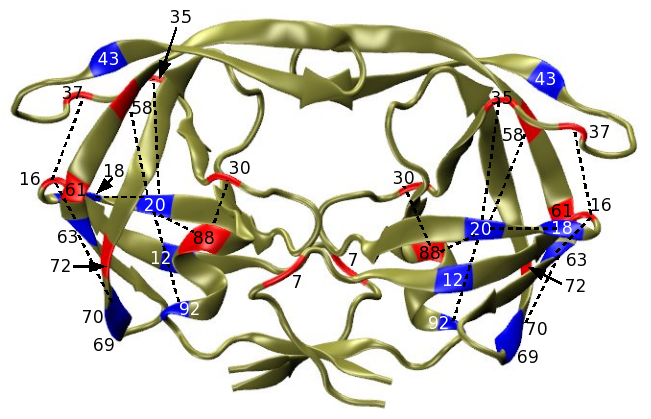

Supplement: Figure S7 — Structure of HIV protease subtype B and the spatial distances between highly correlated pairs. The backbone structure of HIV protease subtype B (PDB ID: 1NH0) is depicted in ribbon format. Similar to Figure 1, the 18 electrostatically active residues are highlighted. Residue positions which have a predominantly negatively charged non-neutral residue in the sequence database are depicted in red. Residues which have a predominantly positively charged non-neutral residue in the database are depicted in blue. Addititionally, the distances between the top 5 most correlated pairs of residues are depicted as dashed lines. The pairs are 30–88, 20–35, 16–63, 18–20 and 20–92. (TIF) [file pcbi.1002675.s007.tif]
