# Supplementary material for: Correlated Electrostatic Mutations Provide a Reservoir of Stability in HIV Protease
Source: PLoS Comput Biol. 2012 Sep 6;8(9):e1002675. doi: 10.1371/journal.pcbi.1002675 (PMC3435258; doi:10.1371/journal.pcbi.1002675)
Supplement: Table S1 — Electrostatic mutation patterns with the highest probabilities under the pair correlation model and the drug combinations they are most strongly associated with. Shown are the top 5 patterns with 2, 3 and 4 electrostatic mutations for which the pair correlation model predicted probability, , is the highest, together with the drug combination they are most significantly associated with. Drug combinations are listed in order of treatment. The test of statistical association between drugs and electrostatic mutation patterns is based on the the Stanford database [31] (SI Methods). The proportion of sequences with the mutation pattern and exposed to a specific drug was compared to the proportion of sequences with the same mutation pattern but exposed to no drugs. The null hypothesis is that that the two proportions are equal, and the p-value to test the significance of this hypothesis is listed alongside the drug combination. NFV: Nelfinavir, IDV: Indinavir, SQV: Saquinavir, RTV: Ritonavir, APV: Amprenavir. The acronym PI, protease inhibitor, is used in the Stanford database when the drug was unknown. The pattern is not significantly associated with any drug combination. (PDF) [file pcbi.1002675.s008.pdf]

Table S1

**Pattern**	$\mathbf{P_2}$	**Drugs**	**p-value**
2 electrostatic mutations			
D30N, N88D	2.7×10^{-2}	NFV	$< 10^{-7}$
K20I, N37D	6.1×10^{-3}	IDV,NFV	$< 10^{-7}$
N37D, H69Q	4.6×10^{-3}	PI	$< 10^{-3}$
N37D, Q61E	2.9×10^{-3}	RTV,SQV,PI	$< 10^{-3}$
Q7E, N37D	2.1×10^{-3}	RTV,PI	$< 10^{-7}$
3 electrostatic mutations			
D30N, N37D, N88D	4.7×10^{-3}	IDV,NFV,RTV	$< 10^{-7}$
K20I, D30N, N88D	3.1×10^{-3}	IDV,NFV,PI	$< 10^{-7}$
D30N, H69Q, N88D	2.7×10^{-3}	IDV,NFV,RTV,SQV	$< 10^{-7}$
D30N, Q61E, N88D	8.1×10^{-4}	NFV	$< 10^{-7}$
Q7E, D30N, N88D	7.4×10^{-3}	NFV	$< 10^{-6}$
4 electrostatic mutations			
K20I, D30N, N37D, N88D	5.5×10^{-4}	IDV,NFV	$< 10^{-7}$
D30N, N37D, H69Q, N88D	3.0×10^{-4}	APV,IDV,NFV,RTV,SQV	$< 10^{-7}$
K20I, D30N, H69Q, N88D	2.4×10^{-4}	NFV,RTV,PI	$< 10^{-7}$
K20I, D30N, E35Q, N88D	2.2×10^{-4}	IDV,NFV	$< 10^{-7}$
D30N, N37D, Q61E, N88D	1.5×10^{-4}	–	–
