# Supplementary material for: Correlated Electrostatic Mutations Provide a Reservoir of Stability in HIV Protease
Source: PLoS Comput Biol. 2012 Sep 6;8(9):e1002675. doi: 10.1371/journal.pcbi.1002675 (PMC3435258; doi:10.1371/journal.pcbi.1002675)
Supplement: Table S2 — Prediction of novel electrostatic mutation patterns. Shown are 25 electrostatic mutation patterns with the highest probabilities under the pair correlation model that are not observed in the Lee database [30]. is the probability of the sequence under the pair correlation model, is the number of times the mutation pattern was found in the Lee database [30], is the number of times the mutation pattern was found in the Stanford database [31]. If the sequence is found in the Stanford database, it may be significantly associated with specific drugs combinations. The drug combinations listed are in order of treatment and have strong p-values of association with the mutation pattern. The test of statistical association between drugs and electrostatic mutation patterns is described in SI Methods. NFV: Nelfinavir, IDV: Indinavir, SQV: Saquinavir, RTV: Ritonavir, APV: Amprenavir. The acronym PI, protease inhibitor, is used in the Stanford database when the drug was unknown. (PDF) [file pcbi.1002675.s009.pdf]

Table S2

**Pattern**	$\mathbf{P_2}$	$\mathbf{N_{LEE}}$	$\mathbf{N_{ST}}$	**Drugs**	**p-value**
H69Q,I72R	1.8×10^{-4}	0	11	APV-IDV-NFV-RTV	$< 10^{-7}$
K20I,N37D,Q58E,Q92K	8.3×10^{-5}	0	5	PI	$< 10^{-5}$
K20I,E34Q,Q58E	7.4×10^{-5}	0	16	PI	$< 10^{-7}$
K20I,L63H,K70E	6.0×10^{-5}	0	4	ATV	$< 10^{-7}$
D30N,H69Q,I72E,N88D	5.3×10^{-5}	0	0	-	-
K20I,D30N,K70E,N88D	5.0×10^{-5}	0	1	PI	4.3×10^{-2}
Q7E,N37D,Q58E	4.6×10^{-5}	0	0	-	-
D30N,I72R,N88D	4.4×10^{-5}	0	0	-	-
Q18H,K43T	4.4×10^{-5}	0	25	LPV-NFV-SQV	$< 10^{-7}$
D30N,L63H,H69Q,N88D	4.2×10^{-5}	0	0	-	-
G16E,I72R	4.1×10^{-5}	0	3	-	-
E34Q,K70E	4.1×10^{-5}	0	4	PI	5.0×10^{-5}
G16E,K20I,N37K	3.8×10^{-5}	0	2	PI	4.1×10^{-3}
Q18H,D30N,N37D,N88D	3.6×10^{-5}	0	2	NFV-PI	$< 10^{-7}$
K20I,D30N,E35Q	3.6×10^{-5}	0	6	NFV-RTV	$< 10^{-7}$
K20I,K70E,Q92K	3.6×10^{-5}	0	0	-	-
T12K,K70T	3.5×10^{-5}	0	18	NFV-PI	7.2×10^{-4}
N37K,K43T,Q61E	3.4×10^{-5}	0	0	-	-
Q18H,K20I,N88D	3.4×10^{-5}	0	3	LPV-RTV-SQV	$< 10^{-7}$
E35Q,Q58E	3.4×10^{-5}	0	21	IDV-LPV-NFV-PI	$< 10^{-7}$
N37D,Q58E,I72E	3.4×10^{-5}	0	3	APV-IDV-NFV-RTV-SQV	$< 10^{-7}$
T12K,N37D,H69Q	3.4×10^{-5}	0	5	-	-
D30N,N37D,L63H,N88D	3.4×10^{-5}	0	1	PI	0.04
G16E,K70E	3.4×10^{-5}	0	6	NFV	4.8×10^{-4}
K20I,D30N,N37D,N88D,Q92K	3.4×10^{-5}	0	1	PI	0.04
