# Supplementary material for: Correlated Electrostatic Mutations Provide a Reservoir of Stability in HIV Protease
Source: PLoS Comput Biol. 2012 Sep 6;8(9):e1002675. doi: 10.1371/journal.pcbi.1002675 (PMC3435258; doi:10.1371/journal.pcbi.1002675)
Supplement: Table S3 — The most statistically deviated pairs of mutations. The 10 most statistically deviated double mutations in the Lee database relative to the independent model. The measure used to test for deviation is where is the joint probability of a double mutation at positions and while is the univariate marginal of a mutation at position . The double mutant charge states and the distance between charges is also listed. (PDF) [file pcbi.1002675.s010.pdf]

Table S3

Residues	Charges	Distance	Enhanced or Suppressed	Deviation
30–88	0,-	6	enhanced	1910
20–35	0,0	11	enhanced	126
16–63	-,+	8	enhanced	118
18–20	+,0	5	enhanced	91
20–92	0,+	21	enhanced	75
63–70	+,-	7	enhanced	56
20–88	0,-	18	enhanced	53
20–58	0,-	20	enhanced	53
16–37	-,-	9	suppressed	48
63–70	+,0	7	enhanced	46
