# Supplementary material for: Correlated Electrostatic Mutations Provide a Reservoir of Stability in HIV Protease
Source: PLoS Comput Biol. 2012 Sep 6;8(9):e1002675. doi: 10.1371/journal.pcbi.1002675 (PMC3435258; doi:10.1371/journal.pcbi.1002675)
Supplement: Text S1 — consists of the supplementary information to the main text. In this supplementary information, we describe our algorithm in greater detail. (PDF) [file pcbi.1002675.s011.pdf]

Supplementary Information: Correlated electrostatic mutations provide a reservoir of stability in HIV protease

Omar Haq[1], Michael Andrec[1,2], Alexandre V. Morozov[3,1] Ronald M. Levy[1,2,*]
1 BioMaPS Institute for Quantitative Biology, Rutgers University, Piscataway, New Jersey, USA
2 Department of Chemistry and Chemical Biology, Rutgers University, Piscataway, New Jersey, USA
3 Department of Physics & Astronomy, Rutgers University, Piscataway, New Jersey, USA
∗ E-mail: Corresponding ronlevy@lutece.rutgers.edu

Materials and Methods

Electrostatic folding free energy calculations

A coarse grained model for the electrostatic free energy contribution to the folding of a given protease sequence was calculated using AGB, an implementation of the pairwise descreening Generalized Born (GB) model that makes use of a parameter-free algorithm to take into account atomic overlaps [1]. Specifically, we computed $\Delta G_e = G_e^{(f)} - G_e^{(u)}$, where $G_e^{(f)}$ and $G_e^{(u)}$ are electrostatic free energies of the folded and unfolded states, respectively. Both $G_e^{(f)}$ and $G_e^{(u)}$ were obtained using

$$G_e \simeq G_{GB} = u_e \sum_i \frac{q_i^2}{B_i} + 2u_e \sum_{i<j} \frac{q_i q_j}{f_{ij}} + \sum_{i<j} \frac{q_i q_j}{\epsilon_{in} r_{ij}} \tag{1}$$

where q_i is the charge of atom i, B_i is its Born radius, $f_{ij} = \sqrt{r_{ij}^2 + B_i B_j \exp(-r_{ij}^2/4B_i B_j)}$ is the GB distance between atoms i and j (r_{ij} is the distance between the charges), and $u_e = 1/2(1/\epsilon_w - 1/\epsilon_{in})$ (ϵ_{in} is the solute dielectric constant and ϵ_w is the solvent dielectric constant) [1]. We used the wild-type crystal structure of HIV protease subtype B (PDB ID 1NH0) [2] to compute $G_e^{(f)}$, and a maximally extended chain A from 1NH0 with backbone dihedral angles set to 180° (except for proline) and sidechain rotamer states set to all-*trans* to compute $G_e^{(u)}$. For both the folded and unfolded structures, unit charges, corresponding to a specific charge signature for the 99 residues, were placed on the most distal carbon atoms. All other side chains atoms are neutralized. Partial charge dipoles of $\pm 0.4e$ are placed on every backbone amide and cabonyl group to preserve helix dipole effects [3].

Posing the inverse problem for the Potts model for sequence probabilities

Our Potts model for correlated electrostatic mutations deals with a reduced sequence length of 18 (from 99 amino acids) and a reduced amino acid alphabet size of 3. For length $N = 18$ and a $Q = 3$ letter alphabet (0, +, -), there are $3^{18} = 387,420,489$ possible sequences or unique charge signatures. For every signature, we wish to calculate the probability of that sequence under two models; an independent model which preserves the database derived univariate marginals and a mean field model (Bethe approximation) which preserves both the database derived univariate and bivariate marginals. We call this the pair correlation model and we refer to the probabilities of a sequence under the independent and pair correlation model as P_1 and P_2 respectively. P_1 probabilities of individual sequences can be obtained by simply taking the product of the observed univariate marginals at each position.

$$P_1(A_1, ..., A_N) = \prod_{i=1}^{N} P_i^{obs}(A_i) \tag{2}$$

where $A_i = \{0, +, -\}$ is one of the three possible charges at position i and $P_i^{obs}(A_i)$ is the observed univariate marginal of charge A_i at position i. All observed univariate and bivariate frequencies are derived from the Lee database [4].

The probability of a sequence under the pair correlation model P_2, on the other hand, is determined by a model which generates sequences that preserve both the observed univariate and bivariate marginals. In order to do so, we fit the sequence signature probabilities to a 3-state Potts model where the Hamiltonian, $\mathcal{H}$ is described by field and coupling parameters, which reflect the mutation frequency at a site and the strength of the statistical coupling between two sites respectively.

$$P_2(A_1, ..., A_N) = \frac{1}{Z}\exp[\mathcal{H}(A_1, ..., A_N)] \tag{3}$$

where $P_2(A_1, ..., A_N)$ is the probability of a sequence of length N consisting of charges A_i at position i which preserves the univariate and bivariate marginals. The Hamiltonian can be defined as

$$\mathcal{H}(A_1, ..., A_N) = \sum_i \lambda_i(A_i) - \sum_{i<j} \lambda_{ij}(A_i, A_j) \tag{4}$$

where $\lambda_i(A_i)$ is the field at position i for charge A_i, $\lambda_{ij}(A_i, A_j)$ is the coupling between charges A_i and A_j at positions i and j and Z is the partition function.

$$Z = \sum_{A_i} \exp[\mathcal{H}(A_1, ..., A_N)] \tag{5}$$

This model is the maximum entropy solution to the probability distribution that matches the single-point and double-point correlations [5]. Note that if there were two possible states at each site instead of three, this Potts model would be equivalent to the famous Ising model, which is widely applied in the study of spin glass systems in statistical physics.

For a system of 18 positions and 3 states, $18 \times 3 = 54$ $\lambda_i(A_i)$ and $\binom{18}{2} \times 3^2 = 1377$ $\lambda_{ij}(A_i, A_j)$ parameters need to be determined to accurately describe the Hamiltonian which preserves the observed marginals. But the paramaters are not independent as we can apply conditions known as gauge constaints which connect the parameters [6]. For each position $\sum_{A_i} \lambda_i(A_i) = 0$ and for each pair of positions $\sum_{A_i} \lambda_{ij}(A_i, A_j) = 0$. This results in two free field parameters per position and four free coupling paramaters for every pair of positions. In this work, following Weigt et al. [6], we have chosen the free parameters so as to maximize the fields and minimize the couplings, on average. In a future communication, we will investigate how the choice of the free parameters affects the information carried by the couplings about spatial proximity.

This inverse problem of determining the fields and couplings, given the univariate and bivariate marginals, is computationally challenging [6]. This problem has been described in the literature as the inverse pairwise Ising problem (for two states) and it is computationally expensive because exact methods to determine the marginals from an initial set of Hamiltonian parameters are slow and therefore iterative procedures to search for the field and coupling parameters for many positions and more than two states is unfeasible. Our own previously described method of fitting pair marginals using iterative proportional fitting (IPF) of log-linear model parameters is slow and may not converge within a desired time frame for the problem at hand [7]. Other proposed methods such as Monte Carlo sampling have been applied on Ising models with a few sites, but may require exponential computational time to converge [8]. The approach we have taken involves iterative inference on a probabilistic graphical model using belief propagation described by Weigt et al. 2009 [6, 9]. The difference between our approach and the approach taken by Weigt and coworkers is that while converging the bivariate marginals, we use a mean field model which includes pair correlations in the Bethe approximation [10]. Applying the Bethe mean field appoximation consistently is just as accurate as using the fluctuation dissipation approach taken by Weigt and coworkers [6, 9]. We will compare the two approaches in a future communication.

Outline of the algorithm

The algorithm iteratively converges upon the field and coupling parameters using gradient descent. The outline of the algorithm is as follows.

1. For a given set of field and coupling parameters, determine the corresponding univariate and bivariate marginals using Belief Propagation and the Bethe mean field approximation.

2. Compare these computed marginals to the observed marginals and update the field and coupling parameters.

3. Repeat steps 1 and 2 until the Bethe mean field approximated univariate and bivariate marginals determined from the updated fields and couplings converge to their observed values from the sequence alignment.

Belief propagation

Belief Propagation (BP) or the Sum-Product algorithm is an iterative procedure applied to efficiently calculate all the marginals in a tree-like graph [11]. It consists of leaf nodes passing messages to their parents, which in turn process these messages and pass them onwards towards the root. The root then sends messages back to its children nodes and so on until the messages eventually reach the leaf nodes. At this point, for acyclic graphs, the messages will converge [12, 13]. For cyclic graphs, this method is approximate but may converge after several cycles of message passing [14–16].

The self-consistent belief propagation equations we have implemented in this paper follow the approach of Weigt et al, 2009 [6]. The probability distribution of the pair correlation model P_2 is given by Equation 3. For this distribution, the corresponding BP message update rule is

$$P_{i\to j}(A_i) \backsim e^{\lambda_i(A_i)} \prod_{k\neq i,j} [\sum_{A_k} e^{-\lambda_{ik}(A_i,A_k)} P_{k\to i}(A_k)] \tag{6}$$

where $P_{i\to j}(A_i)$ is the local message passed from node i to node j. This message is a function of the field at i and the product of all incoming messages from the neighbors of i, not including j. The BP propagation messages are passed locally between nodes with random initial values for the messages. Updates are made and the process is repeated until the messages converge. The proportionality constant is such that the messages at a site sum to 1. Once the messages have converged, marginals are evaluated by taking the product of the field at a site with all the incoming messages to that site

$$P_i(A_i) \backsim e^{\lambda_i(A_i)} \prod_{k\neq i} [\sum_{A_k} e^{-\lambda_{ik}(A_i,A_k)} P_{k\to i}(A_k)] \tag{7}$$

Since our implementation of the network is a completely connected undirected graph, with all nodes interconnected to one another, belief propagation is not guaranteed to converge [12,16,17]. However belief propagation on cyclic graphs, called loopy belief propagation, may closely approximate the solutions after several iterations [14–16].

For our problem, the marginals are known quantities and it is the fields and couplings that we wish to find. Therefore, we actually have an inverse problem; we need to find the fields and couplings given the marginals. This can be achieved by taking the ratios of Equations 6 and 7, a trick described by Weigt et al. 2009 [6, 9], thus allowing us to write the message from i to j in terms of the known marginal at i.

$$\begin{aligned} \frac{P_{i\to j}(A_i)}{P_i(A_i)} &= \frac{e^{\lambda_i(A_i)} \prod_{k\neq i,j} [\sum_{A_k} e^{-\lambda_{ki}(A_k,A_i)} P_{k\to i}(A_k)]}{e^{\lambda_i(A_i)} \prod_{k\neq i} [\sum_{A_k} e^{-\lambda_{ki}(A_k,A_i)} P_{k\to i}(A_k)]} \\ P_{i\to j}(A_i) &= \frac{P_i(A_i)}{\sum_{A_j} e^{-\lambda_{ij}(A_i,A_j)} P_{j\to i}(A_j)]} \end{aligned} \tag{8}$$

Equation 8 can be used to force the univariate marginals estimated by BP to be the observed marginals. As a result, the field parameters never require updating; once the messages converge, the fields can be explicitly calculated using Equation 7. In other words, the univariate marginals are always conserved.

On the other hand, the predicted bivariate marginals need to match the observed bivariate marginals. This can be approximated by the following equation:

$$P_{ij}^{bethe}(A_i, A_j) = \frac{\exp[\lambda_{ij}(A_i, A_j)]P_{i\to j}(A_i)P_{j\to i}(A_j)}{Z} \tag{9}$$

where A_i and A_j are the mutations at positions i and j, $\lambda_{ij}(A_i, A_j)$ is the statistical coupling parameter between i and j, $P_{i\to j}(A_i)$ is the message passed from i to j, $P_{j\to i}(A_j)$ is the message passed from j to i and Z is the partition function. This equation has been proven by Yedidia and coworkers to be mathematically equivalent to the Bethe approximation, a mean field model, and is what we apply in our code to approximate the bivariate marginals in our system [12, 15, 17].

Algorithm in detail

1. Initialization: Set all $\lambda_{ij}(A_i, A_j) = 0$ and all $\lambda_i(A_i) = c_i + \ln P_i(A_i)$, where c_i is a normalization constant for the gauge constraints which were described earlier.

2. Update messages using Equation 8 for all pairs of residues iteratively until the belief propagation messages converge.

3. Update bivariate marginals $P_{ij}^{bethe}(A_i, A_j)$ using the Bethe approximation (Equation 9).

4. Compare $P_{ij}^{bethe}(A_i, A_j)$ to $P_{ij}^{obs}(A_i, A_j)$, which is the database derived frequency of a double mutation. If the couplings have converged, then stop. If the couplings have not converged by a desired amount, update $\lambda_{ij}(A_i, A_j)$ as follows

$$\triangle\lambda_{ij}(A_i, A_j) = -\epsilon[(P_{ij}^{obs}(A_i, A_j) - P_{ij}^{bethe}(A_i, A_j)] \tag{10}$$

where ϵ is the gradient descent step size, set to 0.0001, and repeat steps 2, 3 and 4 until the pair probabilities converge.

Sampling sequences from probability distributions

Several tests of the finite size effects require us to sample sequences drawn from the probability distribution described by either the independent model or the pair correlation model. To randomly sample sequences from these models, we make use of the inverse transform sampling algorithm. In the first step of this algorithm, a cumulative distribution function (CDF) is constructed from the modelled probabilities for a subset of sequences, for example all sequences with 6 mutations. Using subsets allows us to reduce the universe of available sequences and enables faster sampling. Random floating point values between 0 and 1, from a random number generator, are then used to inversely map the CDF back to a specific, randomly picked sequence. Quantile functions for this inverse mapping are saved in a lookup table to allow for efficient sampling. The samples drawn are independent and uncorrelated; hence no burn-in time is required. In effect, we use a Gibbs sampler, i.e. sequences are drawn according to their probabilities under the model and all draws are accepted.

Associating mutation patterns with protease inhibitors

A drug-annotated sequence alignment consisting of 38,420 HIV protease isolates of multiple subtypes was downloaded from the Stanford HIV database on April 7th, 2010 [18]. This dataset was used determine which inhibitors are significantly associated with specific electrostatic mutations patterns. Since multiple isolates in this database are associated with a single patient, only the most recent subtype B isolate for each patient was extracted, leaving 13,286 protease sequences. Upon examination, many sequences come from patients undergoing antiretroviral therapy with one or more protease inhibitors. However, the majority of sequences come from patients who have not been exposed to any drugs. The difference in the

proportion of sequences with a particular mutation pattern in the drug-naive cohort as compared to the proportion of sequences with the same mutation pattern but exposed to a specific drug cocktail, can be used as a measure of association between that drug cocktail and the mutation pattern.

To do find significant associations, the sequence alignment was converted into strings of charge states "n", "-" and "+" as described above, and used to calculate p_{drug}, the proportion of sequences with a unique mutation pattern and exposed to a particular set of drugs, and p_{naive}, the proportion of sequences with the same mutation pattern but exposed to no drugs. A pooled sample proportion t-test was then performed to determine the significance of association for a drug combination with a group of mutations, with the z-score for the null hypothesis of $p_{drug} - p_{naive} = 0$ given by

$$z = \frac{p_{drug} - p_{naive}}{SE}.$$

The standard error, SE is

$$SE = \sqrt{p(1-p)(1/n_1 + 1/n_2)},$$

where $p = (p_{drug}\, n_{drug} + p_{naive}\, n_{naive})/(n_{drug} + n_{naive})$ is the pooled sample proportion, n_{drug} is the number of sequences exposed to the particular combination of drugs, and n_{naive} is the number of sequences not exposed to drugs.

References

1. Gallicchio E, Levy RM (2004) AGBNP: An analytic impicit solvent model suitable for molecular dynamics simulations and high-resolution modeling. J Comput Chem 25: 479–499.

2. Kozisek M, Bray J, Rezacova P, Saskova K, Brynda J, et al. (2007) Molecular analysis of the HIV-1 resistance development: Enzymatic activities, crystal structures, and thermodynamics of nelfinavir-resistant HIV protease mutants. J Mol Biol 374: 1005–1016.

3. Sheridan RP, Levy RM, Salemme FR (1982) Alpha-helix dipole model and electrostatic stabilization of 4-alpha-helical proteins. Proc Nat Acad Sci 79: 4545–4549.

4. Chen L, Perlina A, Lee C (2004) Positive selection detection in 40,000 human immunodeficiency virus (HIV) type 1 sequences automatically identifies drug resistance and positive fitness mutations in HIV protease and reverse transcriptase. J Virol 78: 3722–3732.

5. Good IJ (1963) Maximum entropy for hypothesis formulation, especially for multidimensional contingency tables. Ann Math Stat 34: 911–934.

6. Weigt M, White RA, Szurmant H, Hoch JA, Hwa T (2009) Identification of direct residue contacts in protein-protein interaction by message passing. Proc Nat Acad Sci 106: 67–72.

7. Haq O, Levy RM, Morozov AV, Andrec M (2009) Pairwise and higher-order correlations among drug-resistance mutations in HIV-1 subtype B protease. BMC Bioinformatics 10: S10.

8. Schneidman E, Berry MJ, Segev R, Bialek W (2006) Weak pairwise correlations imply strongly correlated network states in a neural population. Nature 440: 1007–1012.

9. Lunt B, Szurmant H, Procaccini A, Hoch JA, Hwa T, et al. (2010) Inference of direct residue contacts in two-component signaling. Meth Enzym 471: 17–41.

10. Bethe HA (1935) Statistical Theory of Superlattices. Proc Roy Soc London 150: 552.

11. Pearl J (1988) Probabilistic Reasoning in Intelligent Systems: Networks of Plausible Inference. Morgan Kaufmann.

12. Yedidia JS, Freeman WT, Weiss Y (2005) Constructing free energy approximations and generalized belief propagation algorithms. IEEE Transactions on Information Theory 51: 2282–2313.

13. Kschischang FR (2001) Factor Graphs and the Sum-Product Algorithm. IEEE Transactions on Information Theory 47: 498–519.

14. Murphy KP, Weiss Y, Jordan MI (1999) Loopy belief propagation for approximate inference: An empirical study. In: Proceedings of the Fifteenth Conference on Uncertainty in AI.

15. Heskes T (2002) Stable fixed points of loopy belief propagation are minima of the bethe free energy. Advances in Neural Information Processing Systems 15: 343–350.

16. Ihler AT, III JWF, SWillsky A (2005) Loopy belief propagation: Convergence and effects of message errors. Journal of Machine Learning Research 6: 905–936.

17. Yedidia JS, Freeman WT, Weiss Y (2000) Generalized belief propagation. In: NIPS 13.

18. Rhee SY, Gonzales MJ, Kantor R, Betts BJ, Ravela J, et al. (2003) Human immunodeficiency virus reverse transcriptase and protease sequence database. Nucl Acids Res 31: 298–303.

Figure Legends

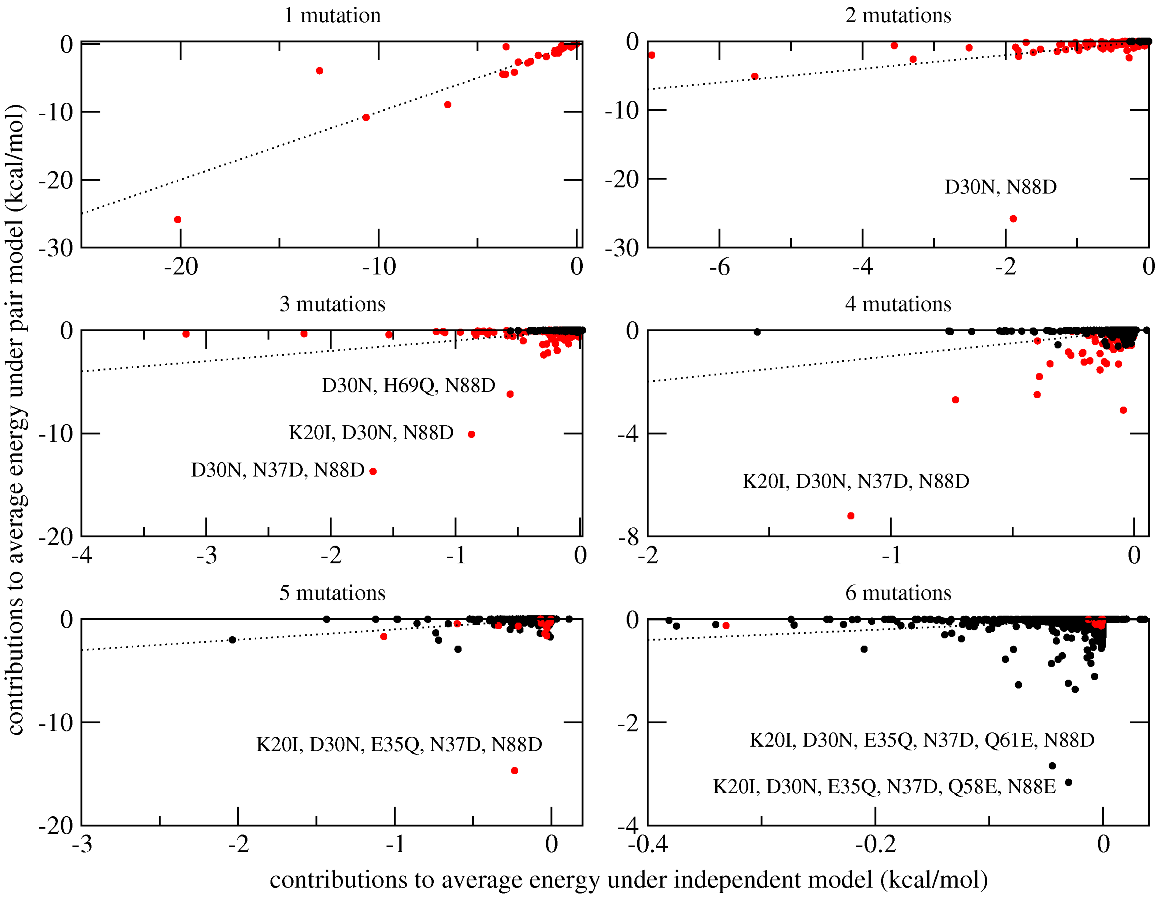

Figure S1. Contribution of individual sequences to the average electrostatic folding energy. Each contribution is given by $\Delta G_e P$, where ΔG_e is the electrostatic folding energy of a given sequence (see Methods) and P is its probabilit y under the independent or pair correlation model conditional upon the number of mutations. Red: mutation patterns observed in the Lee database [4], black: mutation patterns not observed in the Lee database. Several outliers are labeled explicitly by their mutation pattern. Mutations are represented as aNb, where N is the residue number and a and b are one of the 3 charged states (+, -, n). The straight line on each diagram is a plot of $x = y$. Sequences below this line have $P_1 < P_2$, resulting in $\Delta G_e P_1 > \Delta G_e P_2$ ($\Delta G_e < 0$). For these sequences, the electrostatic stabilization is greater under the pair correlation model than under the independent m odel.

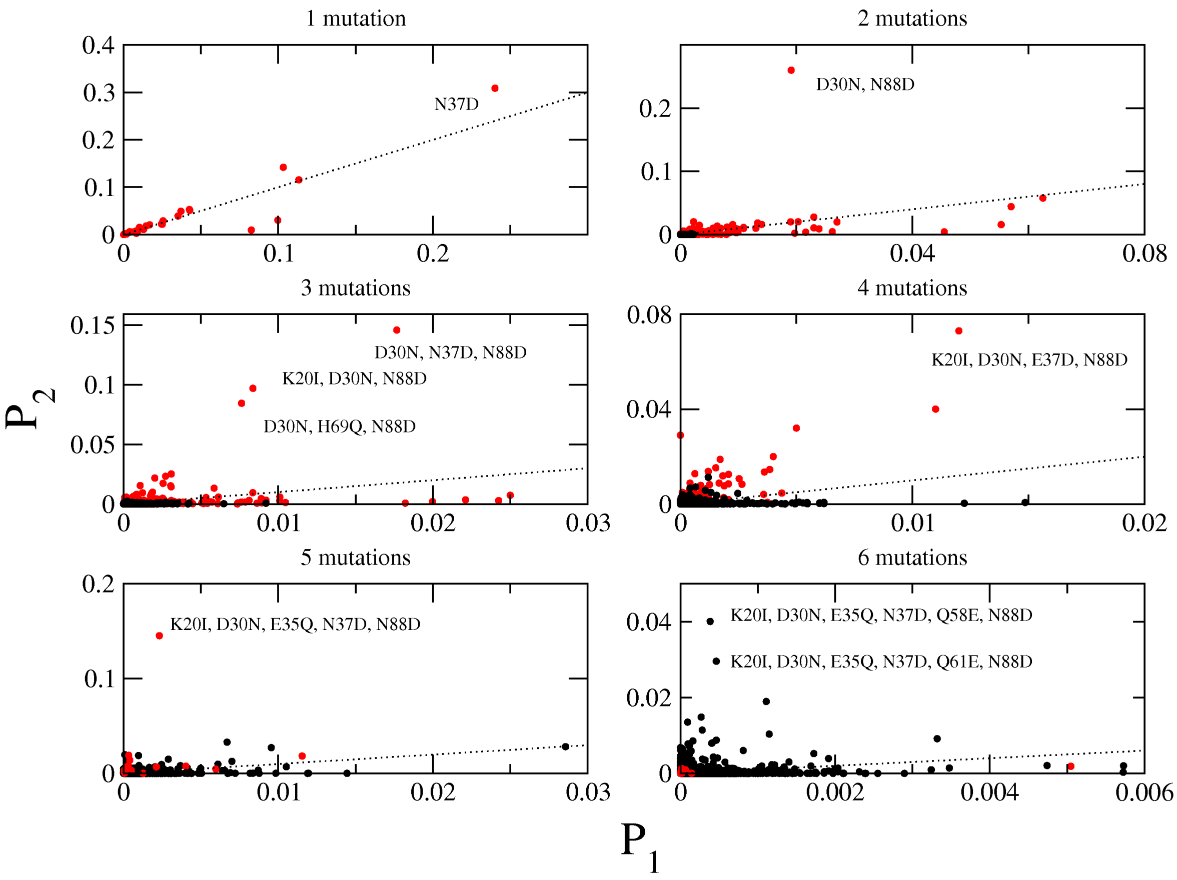

Figure S2. Comparison of sequence probabilities under the independent and pair correlation model. The probability of a given sequen ce under the pair correlation model, P_2, is plotted against the probability of the same sequence under the independent mo del, P_1, for all sequences with 1 through 6 electrostatic mutations. Both independent and pair correlation model probabil ities are renormalized and are conditional upon the number of mutations. Red: mutation patterns observed in the Lee database citeChen:2004ao, black: mutation patterns not observed in the Lee database. Several outliers are labeled explicitly by their mutation pattern. Mutations are represented as aNb, where N is the residue number and a and b are one of the 3 cha rged states (+,-, n). The straight line on each diagram is a plot of $x = y$. Sequences below this line have $P_1 < P_2$.

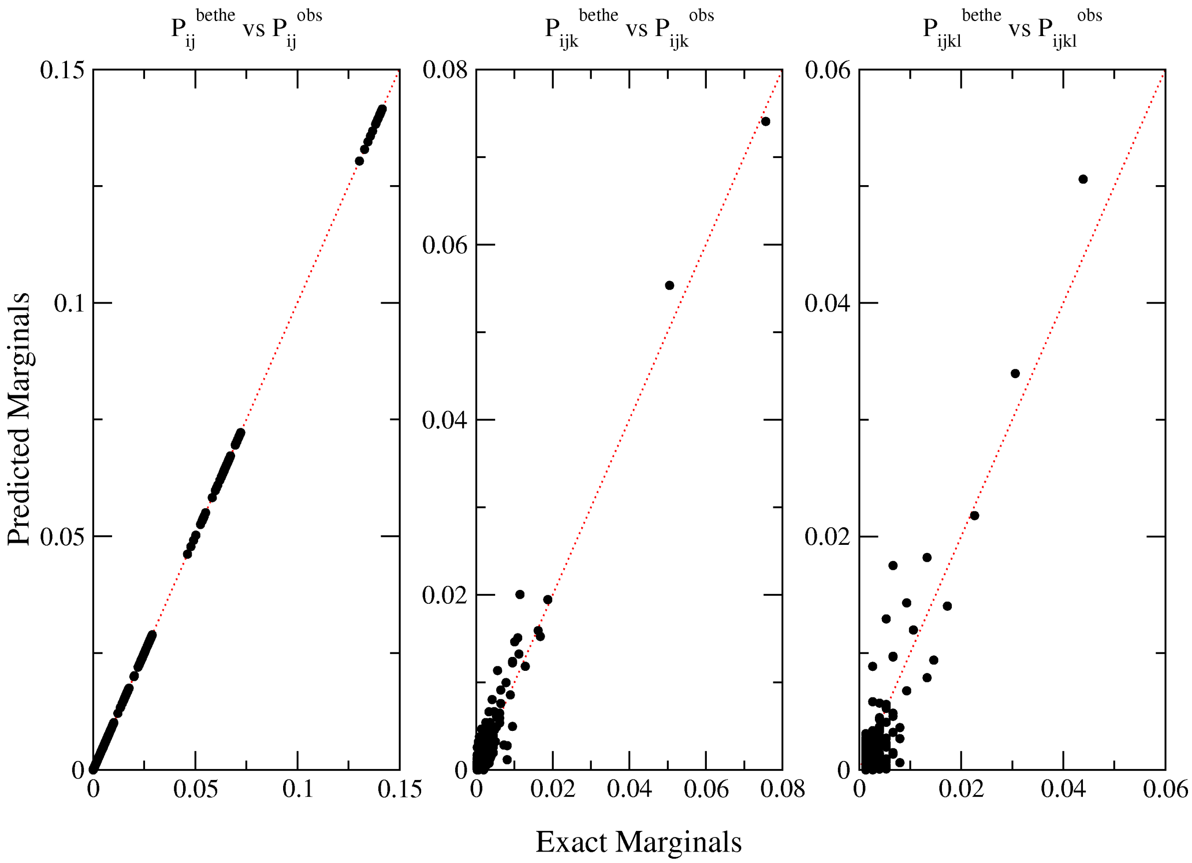

Figure S3. Comparison between the observed and predicted mutivariate marginals for 2, 3 and 4 mutations. Predicted marginals determined using belief propagation in the Bethe approximation are plotted against the observed marginals for sets of 2, 3, and 4 mutations. The correlation between P_{ij}^{bethe} and P_{ij}^{obs} is 1.00. The correlation between P_{ijk}^{bethe} and P_{ijk}^{obs} is 0.98. The correlation between P_{ijkl}^{bethe} and P_{ijkl}^{obs} is 0.90.

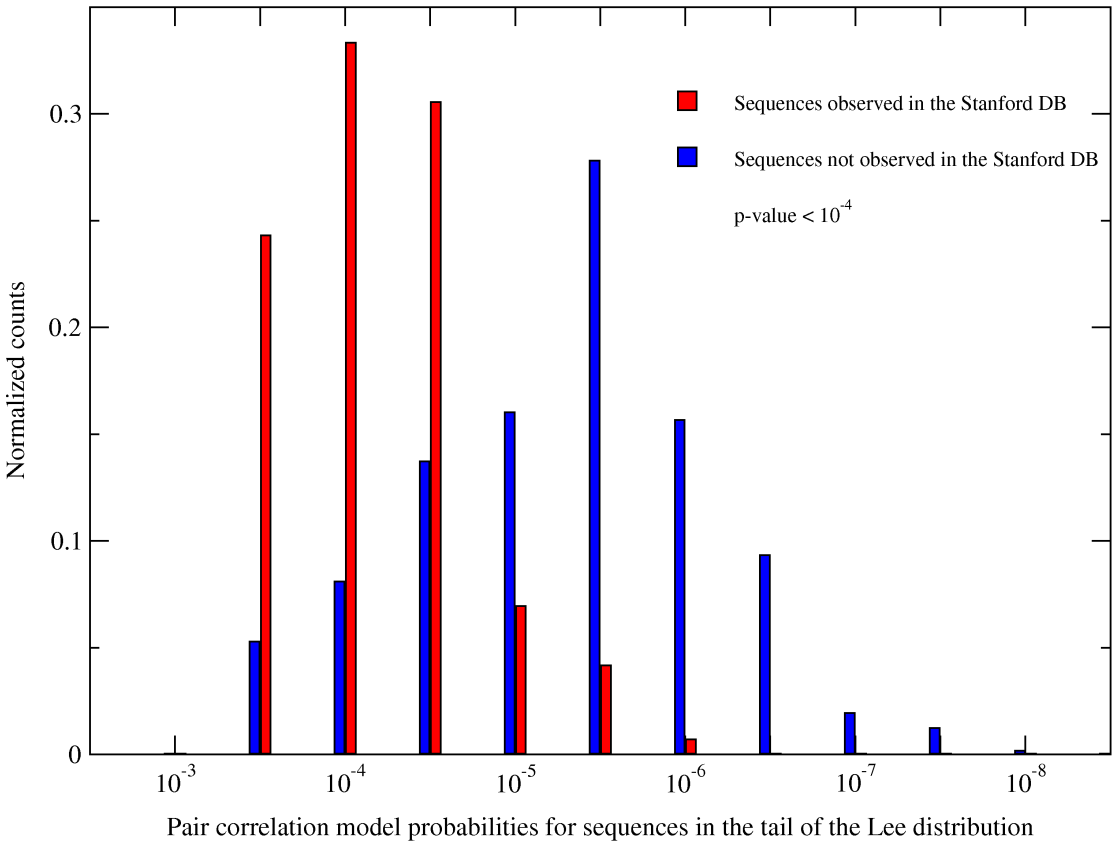

Figure S4. Distribution of pair correlation model probabilities for sequences in the tail of the Lee distribution that are observed (red) or unobserved (blue) in the Stanford database. The histogram in red is the distribution of pair correlation model probabilities for sequences found in the tail of the Lee database that also exist in the Stanford database. The histogram in blue is the distribution of pair correlation model probabilities for sequences that are not observed in the Stanford database. The null hypothesis which states that the means of these two distributions are equal, has a low p-value of $< 10^{-4}$, indicating that the null hypothesis must be rejected. Therefore, the difference between the means of these two distributions is statistically significant.

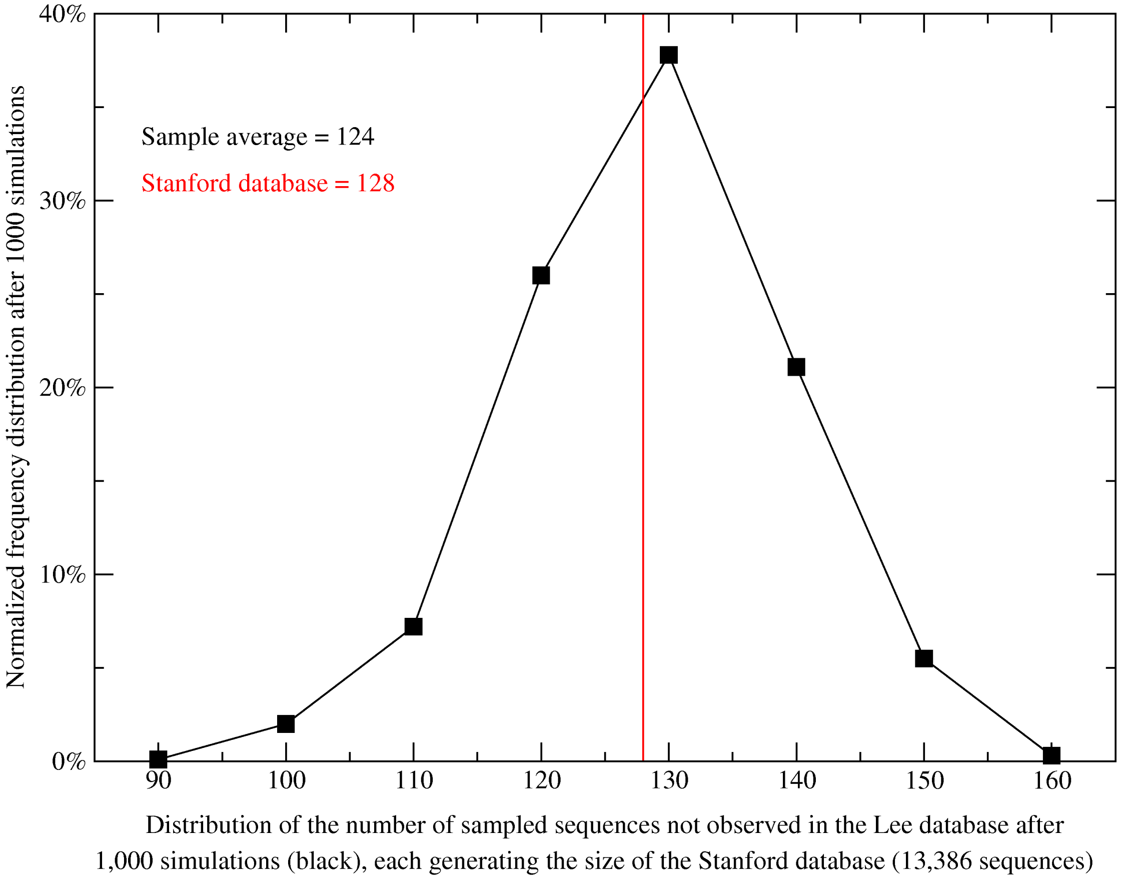

Figure S5. Distribution of the number of sampled sequences not observed in the Lee database. 13,286 sequences, corresponding to the size of the Stanford database, were randomly sampled from the probability distribution described by the pair correlation model. The distribution of the number of sequences not observed in the Lee database for each of the 1,000 simulations, is plotted as a frequency distribution. The sample average for this distribution is 124.2 and the standard deviation is 10.6. The actual number of sequences in the Stanford database that are not observed in the Lee database is 128 (plotted as a straight red line), a number which lies well within 1 standard deviation of the sample mean.

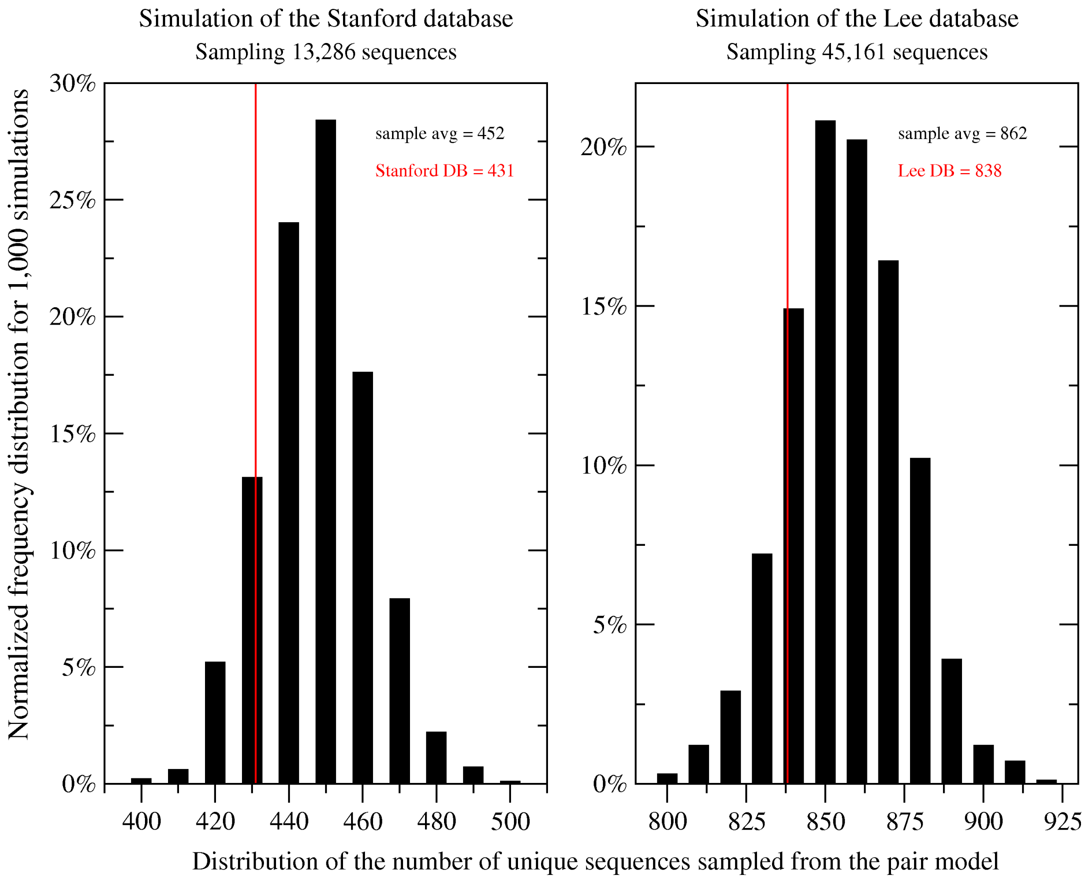

Figure S6. Distribution of the number of unique sequences for sample sizes equal to the size of Stanford and Lee databases. 13,286 and 45,161 sequences, corresponding to the sizes of the Stanford and Lee databases, were each randomly sampled from the probability distribution described by the pair correlation model. The distribution of the number of unique sequences for 1,000 simulations for both sampling distributions is plotted as a histogram. The sample average for the Stanford-sized sample distribution is 452.9 and the standard deviation is 14.3. The sample average for the Lee-sized sample distribution is 862.1 and the standard deviation is 18.7. The number of unique sequences in the Stanford database is 431 while the number of unique sequences in the Lee database is 828, both of which lie within 1.4 standard deviations of their respective sample means.

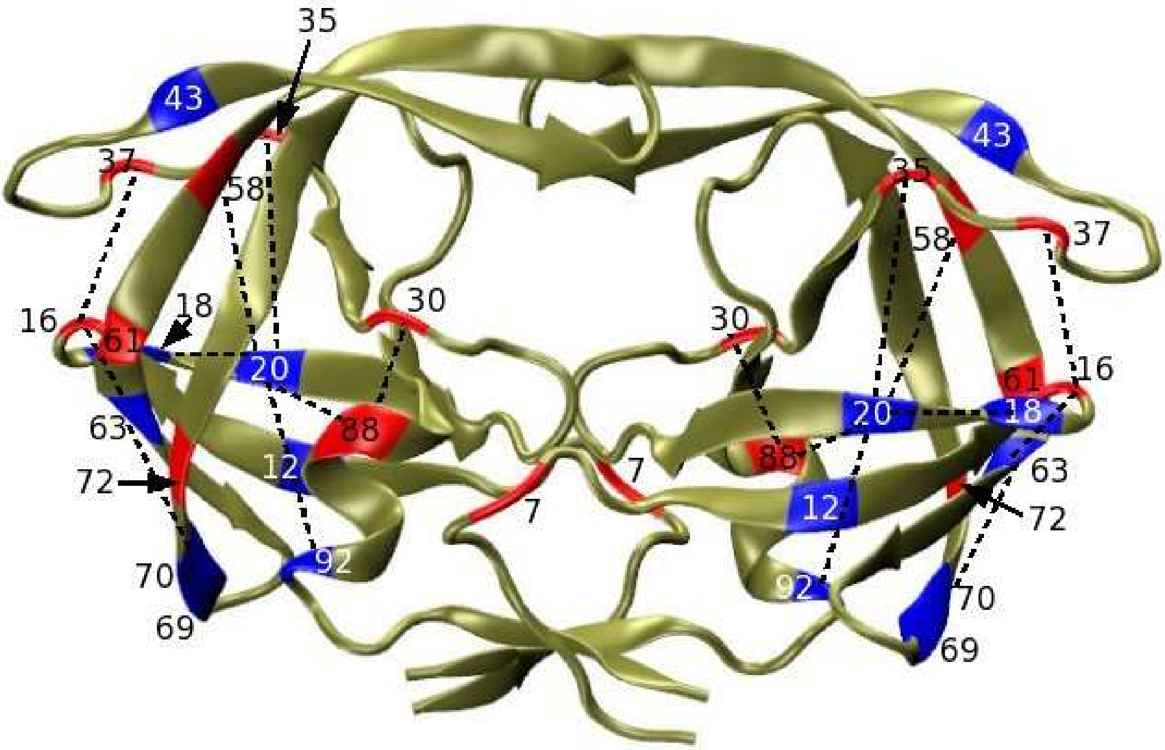

Figure S7. Structure of HIV protease subtype B and the spatial distances between highly correlated pairs. The backbone structure of HIV protease subtype B (PDB ID: 1NH0) is depicted in ribbon format. Similar to Figure 1, the 18 electrostatically active residues are highlighted. Residue positions which have a predominantly negatively charged non-neutral residue in the sequence database are depicted in red. Residues which have a predominantly positively charged non-neutral residue in the database are depicted in blue. Addititionally, the distances between the top 5 most correlated pairs of residues are depicted as dashed lines. The pairs are 30–88, 20–35, 16–63, 18–20 and 20–92.

Tables

Table S1. Electrostatic mutation patterns with the highest probabilities under the pair correlation model and the drug combinations they are most strongly associated with. Shown are the top 5 patterns with 2, 3 and 4 electrostatic mutations for which the pair correlation model predicted probability, P_2, is the highest, together with the drug combination they are most significantly associated with. Drug combinations are listed in order of treatment. The test of statistical association between drugs and electrostatic mutation patterns is based on the the Stanford database [18] (SI Methods). The proportion of sequences with the mutation pattern and exposed to a specific drug was compared to the proportion of sequences with the same mutation pattern but exposed to no drugs. The null hypothesis is that that the two proportions are equal, and the p-value to test the significance of this hypothesis is listed alongside the drug combination. NFV: Nelfinavir, IDV: Indinavir, SQV: Saquinavir, RTV: Ritonavir, APV: Amprenavir. The acronym PI, protease inhibitor, is used in the Stanford database when the drug was unknown. The $D30N, N37D, Q61E, N88D$ pattern is not significantly associated with any drug combination.

Pattern	$\mathbf{P_2}$	Drugs	p-value
2 electrostatic mutations			
D30N, N88D	2.7×10^{-2}	NFV	$< 10^{-7}$
K20I, N37D	6.1×10^{-3}	IDV,NFV	$< 10^{-7}$
N37D, H69Q	4.6×10^{-3}	PI	$< 10^{-3}$
N37D, Q61E	2.9×10^{-3}	RTV,SQV,PI	$< 10^{-3}$
Q7E, N37D	2.1×10^{-3}	RTV,PI	$< 10^{-7}$
3 electrostatic mutations			
D30N, N37D, N88D	4.7×10^{-3}	IDV,NFV,RTV	$< 10^{-7}$
K20I, D30N, N88D	3.1×10^{-3}	IDV,NFV,PI	$< 10^{-7}$
D30N, H69Q, N88D	2.7×10^{-3}	IDV,NFV,RTV,SQV	$< 10^{-7}$
D30N, Q61E, N88D	8.1×10^{-4}	NFV	$< 10^{-7}$
Q7E, D30N, N88D	7.4×10^{-3}	NFV	$< 10^{-6}$
4 electrostatic mutations			
K20I, D30N, N37D, N88D	5.5×10^{-4}	IDV,NFV	$< 10^{-7}$
D30N, N37D, H69Q, N88D	3.0×10^{-4}	APV,IDV,NFV,RTV,SQV	$< 10^{-7}$
K20I, D30N, H69Q, N88D	2.4×10^{-4}	NFV,RTV,PI	$< 10^{-7}$
K20I, D30N, E35Q, N88D	2.2×10^{-4}	IDV,NFV	$< 10^{-7}$
D30N, N37D, Q61E, N88D	1.5×10^{-4}	–	–

Table S2. Prediction of novel electrostatic mutation patterns. Shown are 25 electrostatic mutation patterns with the highest probabilities under the pair correlation model that are not observed in the Lee database [4]. P_2 is the probability of the sequence under the pair correlation model, N_{LEE} is the number of times the mutation pattern was found in the Lee database [4], N_{ST} is the number of times the mutation pattern was found in the Stanford database [18]. If the sequence is found in the Stanford database, it may be significantly associated with specific drugs combinations. The drug combinations listed are in order of treatment and have strong p-values of association with the mutation pattern. The test of statistical association between drugs and electrostatic mutation patterns is described in SI Methods. NFV: Nelfinavir, IDV: Indinavir, SQV: Saquinavir, RTV: Ritonavir, APV: Amprenavir. The acronym PI, protease inhibitor, is used in the Stanford database when the drug was unknown.

**Pattern**	$\mathbf{P_2}$	$\mathbf{N_{LEE}}$	$\mathbf{N_{ST}}$	**Drugs**	**p-value**
H69Q,I72R	1.8×10^{-4}	0	11	APV-IDV-NFV-RTV	$< 10^{-7}$
K20I,N37D,Q58E,Q92K	8.3×10^{-5}	0	5	PI	$< 10^{-5}$
K20I,E34Q,Q58E	7.4×10^{-5}	0	16	PI	$< 10^{-7}$
K20I,L63H,K70E	6.0×10^{-5}	0	4	ATV	$< 10^{-7}$
D30N,H69Q,I72E,N88D	5.3×10^{-5}	0	0	-	-
K20I,D30N,K70E,N88D	5.0×10^{-5}	0	1	PI	4.3×10^{-2}
Q7E,N37D,Q58E	4.6×10^{-5}	0	0	-	-
D30N,I72R,N88D	4.4×10^{-5}	0	0	-	-
Q18H,K43T	4.4×10^{-5}	0	25	LPV-NFV-SQV	$< 10^{-7}$
D30N,L63H,H69Q,N88D	4.2×10^{-5}	0	0	-	-
G16E,I72R	4.1×10^{-5}	0	3	-	-
E34Q,K70E	4.1×10^{-5}	0	4	PI	5.0×10^{-5}
G16E,K20I,N37K	3.8×10^{-5}	0	2	PI	4.1×10^{-3}
Q18H,D30N,N37D,N88D	3.6×10^{-5}	0	2	NFV-PI	$< 10^{-7}$
K20I,D30N,E35Q	3.6×10^{-5}	0	6	NFV-RTV	$< 10^{-7}$
K20I,K70E,Q92K	3.6×10^{-5}	0	0	-	-
T12K,K70T	3.5×10^{-5}	0	18	NFV-PI	7.2×10^{-4}
N37K,K43T,Q61E	3.4×10^{-5}	0	0	-	-
Q18H,K20I,N88D	3.4×10^{-5}	0	3	LPV-RTV-SQV	$< 10^{-7}$
E35Q,Q58E	3.4×10^{-5}	0	21	IDV-LPV-NFV-PI	$< 10^{-7}$
N37D,Q58E,I72E	3.4×10^{-5}	0	3	APV-IDV-NFV-RTV-SQV	$< 10^{-7}$
T12K,N37D,H69Q	3.4×10^{-5}	0	5	-	-
D30N,N37D,L63H,N88D	3.4×10^{-5}	0	1	PI	0.04
G16E,K70E	3.4×10^{-5}	0	6	NFV	4.8×10^{-4}
K20I,D30N,N37D,N88D,Q92K	3.4×10^{-5}	0	1	PI	0.04

Table S3. The most statistically deviated pairs of mutations. The 10 most statistically deviated double mutations in the Lee database relative to the independent model. The measure used to test for deviation is $dev(i,j) = \frac{(P_{ij}(M,M) - P_i(M)P_j(M))^2}{P_{ij}(M,M)}$ where $P_{ij}(M,M)$ is the joint probability of a double mutation at positions i and j while $P_i(M)$ is the univariate marginal of a mutation at position i. The double mutant charge states and the distance between charges is also listed.

Residues	Charges	Distance	Enhanced or Suppressed	Deviation
30–88	0,-	6	enhanced	1910
20–35	0,0	11	enhanced	126
16–63	-,+	8	enhanced	118
18–20	+,0	5	enhanced	91
20–92	0,+	21	enhanced	75
63–70	+,-	7	enhanced	56
20–88	0,-	18	enhanced	53
20–58	0,-	20	enhanced	53
16–37	-,-	9	suppressed	48
63–70	+,0	7	enhanced	46
